# Supplementary material for: Programmable memristors with two-dimensional nanofluidic channels
Source: Nat Commun. 2025 Jul 30;16:7008. doi: 10.1038/s41467-025-61649-6 (PMC12311041; doi:10.1038/s41467-025-61649-6)
Supplement: Supplementary file 1 — Supplementary Information [file 41467_2025_61649_MOESM1_ESM.pdf]

Supplementary Materials for

Programmable memristors with two-dimensional nanofluidic channels

Abdulghani Ismail[1,2], Gwang-Hyeon Nam[1,2], Aziz Lokhandwala,[1,2] Siddhi Vinayak Pandey,[1,2] Kalluvadi Veetil Saurav,[2,3] Yi You,[1,2] Hiran Jyothilal,[1,2] Solleti Goutham[1,2], Ravalika Sajja[1,2], Ashok Keerthi[2,3,4], Boya Radha[1,2,4*]

[1]Department of Physics and Astronomy, School of Natural Sciences, The University of Manchester, Manchester M13 9PL, United Kingdom

[2]National Graphene Institute, The University of Manchester, Manchester M13 9PL, United Kingdom

[3]Department of Chemistry, School of Natural Sciences, The University of Manchester, Manchester M13 9PL, United Kingdom

[4]Photon Science Institute, The University of Manchester, Manchester M13 9PL, United Kingdom

* Correspondence to be addressed to: radha.boya@manchester.ac.uk

Contents

Section 1: Minimal model simulations 2

1. Theoretical Model of Nanofluidic Memristors: A Unified Framework for four Distinct Memristive Behaviors 2
2. Mathematical Framework 2
3. Memory Loop Behaviors and Conditions 4
4. Experimental Parameters and Loop Simulation 5

Section 2: Supplementary figures 6

1. Nanochannel device geometry 6
2. Process flow of device fabrication 6
3. Conductance-voltage curves of nanochannel memristors 7
4. Voltage-dependence of different memristive effects 8

5. Frequency-dependence of different memristive effects 11
6. Surface charge inversion 13
7. Change of memristive style by concentration or pH 18
8. Memristor variation with channel height and electrolyte concentration: 22
9. Effect of electrolyte salt type 25
10. Types of observed saturation memristors 28
11. Endurance of memristors 29
12. Successive conductance strengthening and weakening 31
13. Relaxation at zero-volt in Wien effect 32
14. Crossing 2 memristor 33
a) Voltage amplitude effect 34
b) Pulse duration effect 35
15. Temperature effect 35
References 36

Section 1: Minimal model simulations

1. Theoretical Model of Nanofluidic Memristors: A Unified Framework for four Distinct Memristive Behaviors

Memristive behaviors observed in 2D nanochannels result from a combination of ion–ion interactions, ion–channel wall interactions, and external polarization resulting in channel entrance depletion. We build upon a minimal model to describe four experimentally observed memristive loops (M1-crossing 1), M2-saturation, M3-crossing 2, and M4-Wien), through a set of dynamical equations. We extend and unify the minimal model originally proposed in a previous article[3]. Our extended model incorporates coupled ion transport, adsorption–desorption kinetics, ionic pairing, and voltage-driven depletion from reservoirs.

2. Mathematical Framework

In the simplest picture, the memristive response of a nanochannel can be described by an internal state variable, representing the fraction of ions actively contributing to conduction. Its time evolution is governed by adsorption-desorption dynamics at the channel walls and/or by the ionic association-dissociation within the channel. If the selectivity of these channels has to be taken into account, this can be two (or more) internal state variables representing the fraction of ions of a given polarity. The contribution of association/dissociation term can be explicit to n or can be made implicit into the F_{ads}/F_{des}. This can be expressed as follows.

$$\frac{dn}{dt} = F_{ads}(n,\sigma,V) - F_{des}(n,\sigma,V) \pm F_{ionic}(n,V) \quad (1)$$

where $\sigma(t)$ denotes the fraction of ions adsorbed on channel walls, F_{ionic} represents the ion-ion interaction term which can depend on voltage $V(t)$ in case of Onsager's second Wien effect, or fractional Wien effect in nanopores, or it could be voltage independent, if ions of different polarity are obeying predator/prey dynamics at low concentrations. The ionic current at each instant depends on the instantaneous internal state as follows:

$$I(t) = G_{int}(n,\ V)\,V(t) \tag{2}$$

The key to obtain different memristive loops lies in how $F_{ads}, F_{des}, F_{ionic}$ and G_{int} respond differently to changes in ionic concentration, association/dissociation dynamics, surface charge, and external voltage polarity and magnitude. Considering these, below we extend our previous minimal model [3] to obtain all 4 memristive loops. Since, the higher valency electrolytes might form polymeric and oligomeric complexes, the discussion will be made for a monovalent electrolyte.

Let c_A and c_B denote the spatial average of concentrations of A^+ and B^- ions in the nanochannel. Similarly, σ_A and σ_B represent the net flux of these ions adsorbed on the channel walls. The nanochannel is connected to asymmetric left and right reservoirs with ion concentrations c_l and c_r, and an external voltage drives ionic motion, modeled via a normalized forcing function $f(t)$. We define, $t = t_{actual} * D/L^2$, where L is the length of channel, typically, $\sim 5\ \mu m$, t_{actual} in seconds and D is the diffusion coefficient. The factor D/L^2 must be considered during memory window calculation for individual switching types [3]. For sake of simplicity, we will work with dimensionless parameters throughout this discussion. Adapting the kinetic approach, the concentration of A^+ and B^- ions within the channel is governed by the following transport equations.

- Transport equations:

$$\dot{c_A} = -c_A + 0.5 * (c_l + c_r) + 0.5 * (c_l - c_r) * f(t) - (\dot{\sigma_A}) \tag{3}$$

$$\dot{c_B} = -c_B + 0.5 * (c_l + c_r) + 0.5 * (c_l - c_r) * f(t) - (\dot{\sigma_B}) \tag{4}$$

These equations describe:

i) Natural decay to equilibrium: First term in equations (3) and (4) indicates the diffusive decay, and second term indicates value at equilibrium.
ii) Ion input from reservoirs modulated by $f(t)$: Third term in equations (3) and (4) indicates the changes in concentration of ionic species with forcing $f(t)$.
iii) Losses due to adsorption onto walls: This is described by the fourth term in equations (3) and (4) which is governed by equations (5) and (6).
iv) Ion-Ion interaction: This incorporated into the fourth term of adsorption/desorption dynamics in equations (5) and (6) and its voltage dependence is governed by equation (7).

- Adsorption/Desorption dynamics:

$$\dot{\sigma_A} = \alpha_A c_A - \beta_B c_B - l_A \sigma_A - \delta_0[f] c_A c_B \tag{5}$$

$$\dot{\sigma_B} = -\beta_B c_B - \alpha_A c_A - l_B \sigma_B + \delta_0[f] c_A c_B \tag{6}$$

$$\delta_0[f] = \frac{\delta}{1+\exp(\phi|f|)} \tag{7}$$

where α_A, β_B are adsorption rates, and l_A, l_B are desorption rates.

- Ionic interaction:

The ionic interaction term is considered in equations (5-7), by assuming that ionic interactions obey predator-prey dynamics within the channel. Since the M4 switching in the fluidic channels is associated with Wien effect, it is reasonable to assume that the strength of this term depends on applied voltage. The voltage or here the forcing dependence of this interaction term is defined as δ_0 in equation 7.

The channel conductance G_{int} is given by:

$$G_{int} = c_A e\mu_A + c_B e\mu_B \tag{8}$$

$$\text{Advection Flux} = G_{int} * f(t) \tag{9}$$

To incorporate, the depletion (M2 loop) in this model, we introduce a depletion factor $\Theta(f)$ which depends on $\gamma\epsilon[0,1]$ defined as follows.

$$\Theta(f) = \exp\left(-\gamma\Omega(f)\right) \tag{10}$$

$\gamma = 0$ when channel is fully depleted, and $\gamma = 1$ when channel depletion is completely absent. Any value between zero and one of this factor will indicate a mixed state pre-set within the channel. This factor enters the above equation as follows.

$$\dot{c_A} = -c_A + \Theta(f,t).\left[0.5 * (c_l + c_r) + 0.5 * (c_l - c_r) * f(t) - (\dot{\sigma_A})\right] \tag{11}$$

$$\dot{c_B} = -c_B + \Theta(f,t).\left[0.5 * (c_l + c_r) + 0.5 * (c_l - c_r) * f(t) - (\dot{\sigma_B})\right] \tag{12}$$

Here, we assume, $\Omega(f) = f^2$, yields:

$$\Theta(f) = e^{-\gamma f^2} \tag{13}$$

The modified advection flux is given as follows:

$$\text{Advection flux} = G(\Theta(f)).f(t) \tag{14}$$

where, G is modified conductance defined from equations (11, 12) in equation (8).

Heuristic Interpretation

- Without forcing, in absence of any interaction terms: $c_A = c_B = \frac{c_l+c_r}{2}$ in the channel, and $c_A = c_B = c_l/2$ in the left reservoir and $c_A = c_B = c_r/2$ in the right reservoir.
- With forcing, without interaction terms: Asymmetric ionic entry leads to rectification depending on $f(t)$, but cannot explain the memory in the desired memory window. The concentration (c_A, c_B) is modified as follows.
  - Left reservoir $\left(\frac{c_l}{2}\left(1 - f(t)\right), \frac{c_l}{2}\left(1 + f(t)\right)\right)$
  - Right reservoir $\left(\frac{c_r}{2}\left(1 + f(t)\right), \frac{c_r}{2}\left(1 - f(t)\right)\right)$
  - nanochannel $\left(\frac{c_l+c_r}{2} + \left(\frac{c_l-c_r}{2}\right)f(t), \frac{c_l+c_r}{2} - \left(\frac{c_l-c_r}{2}\right)f(t)\right)$
- Adsorption/desorption dynamics of $A^+$and B^- ions in eq (5-7) can be understood as follows:
  - 1st term: Higher the concentration of A^+ ion within the channel, more chances are for A^+ ion to be adsorbed onto the surface of channel.
  - 2nd term: Higher the concentration B^- ions, lesser effective it will be for A^+ ions to get adsorbed.
  - 3rd term: The adsorbed ions within the channel will desorb with rate l_A/l_B.
  - 4th term: Considers the predator-prey type ionic interaction of opposite polarity ions.

3. Memory Loop Behaviors and Conditions

Each of the four observed memory types corresponds to specific parameter regimes within this unified model.

- M1 (Crossing 1):
  - Dominated by strong A^{+} adsorption/desorption.
  - $\alpha_A, l_A \gg \delta; \beta_B, l_B \ll \delta; \alpha_A > \delta > \beta_B$.
  - B^{-} adsorption minimal.
  - Results in bipolar hysteresis with crossing direction reversed compared to M3.
  - γ=0.
  - Forcing frequency used: 60 mHz.
  - Asymmetry: $c_l = 10, c_r = 100$.
- M3 (Crossing 2):
  - Similar to M1 but with inverted adsorption rate hierarchy.
  - $\alpha_A, l_B \ll \delta; \beta_B, l_A \gg \delta; \beta_B > \delta > \alpha_A$.
  - Rectification favors the opposite voltage polarity.
  - γ=0.
  - Forcing frequency used: 75 mHz.
  - Asymmetry: $c_l = 10, c_r = 100$.
- M4 (Wien-type):
  - Dominated by ion-ion interaction (Bjerrum pairing).
  - $\alpha_A \sim \delta; l_A, l_B, \beta_B < \delta; l_B < \beta_B, l_A$.
  - Yields unipolar nonlinear hysteresis symmetric in voltage.
  - γ=0
  - Forcing frequency used: 35 mHz.
- M2 (Saturation type):
  - Occurs when ion supply from the external reservoir to the channel becomes limiting at high voltage with selectivity of the nanochannel akin to conditions of M1.
  - γ=1, reservoir depletion
  - Captures saturation and possible negative differential resistance.
  - Forcing frequency used: 0.1 Hz.

4. Experimental Parameters and Loop Simulation

Although, clear switching trends are presented with zero-point initial conditions for $c_A, c_B, \sigma_A, \sigma_B$, i.e. $c_A[t=0] = c_B[t=0] = \sigma_A[t=0] = \sigma_B[t=0] = 0$, appropriate values of them were chosen (available in the code) to have zero crossing condition intact. In Figure 5, a few points were removed for values of f in range $0 \to 0.12$, to enable comparison among different switching mechanisms. This also distinguishes the presented system from an ideal memristor, as for an ideal memristor, irrespective of value of state variable at any times, at zero voltage current is zero. The observed loop shapes arise from solving the coupled ordinary differential equations for $c_A, c_B, \sigma_A, \sigma_B$ under time-dependent forcing $f(t)$. The model shows 4 (M1- M4) distinct loop shapes in both advection flux versus f and conductance G versus f, in line with experimental observations.

This unified model captures the diverse spectrum of nanofluidic memristive responses through set of coupled equations. The memristive loop type depends critically on the relative magnitude of adsorption, desorption, and ion-pairing rates. The minimal model, when extended with voltage-induced depletion effects, also allows inclusion of saturation (M2). Together, these mechanisms explain the four principal types of memristive loops and provide the predictive power for designing programmable ionic devices using 2D nanofluidic channels.

The shortcomings of this model is with the assumption that only pairwise ionic interactions are dominant which is inspired from the Bjerrum hypothesis and predator-prey model. Together with this, the model is presented for the monovalent electrolytes, and might not be straightforward to extend for multivalent electrolytes. Further development of this model could include multiple ion-ion interactions. Additionally, the functional form introduced in equations (7), (10) and (13) is arbitrary

and is based on the rationale that at higher voltages the effect of depletion of channels (or crowding of ions near the entrance) is less prominent, which could be improved in future studies.

Section 2: Supplementary figures

1. Nanochannel device geometry

Our 2D nanochannels are symmetrical along the ion path plane, resembling a parallelepiped with a constant cross-section. These slit-like nanochannels allow free ion movement in two dimensions (length and width) while restricting it in height, which could range from values of 0.68 nm to several nm.

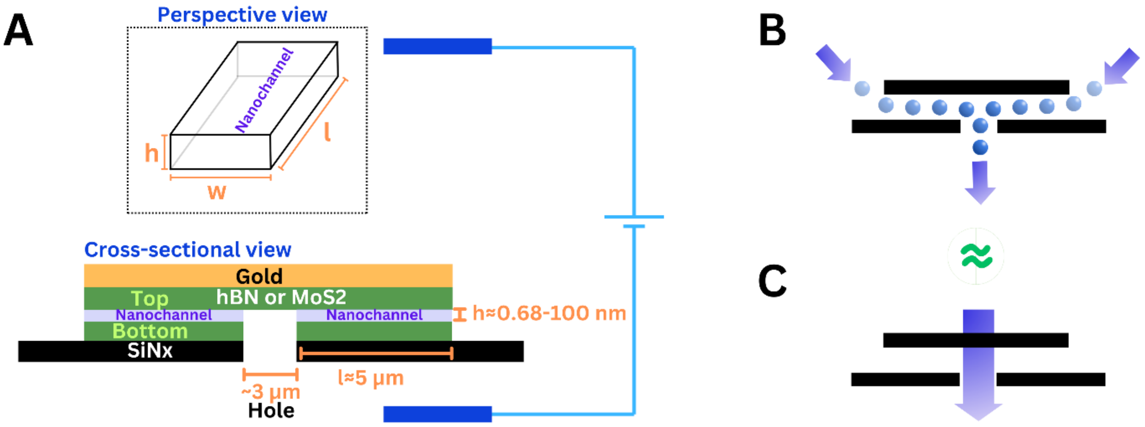

Supplementary Figure 1: Schematic of the geometry of the nanocapillary device. A) 2D cross-sectional view of the device illustrates the dimensions and walls of the nanochannels. The device comprises ~200 parallel nanochannels, each shaped as a parallelepiped with a length in the micrometer range (top inset). The spacer's height determines the nanochannel height (ranging from 0.68 to 100 nm), while the distance between the graphene nanoribbons sets the nanochannel width at ~120 nm. B) The ion flow direction from channel side to the hole side. The flow can be either from hole side or channel side depending on the gradient and the driving voltage. C) Simplified depiction of the same flow direction as in B, is used in some of the figures.

2. Process flow of device fabrication

The device's fabrication method is depicted in Supplementary Figure 2 below. The figure shows the steps for fabricating MoS_2 device, however, the same steps could be used to fabricate the hBN devices with a graphene spacer.

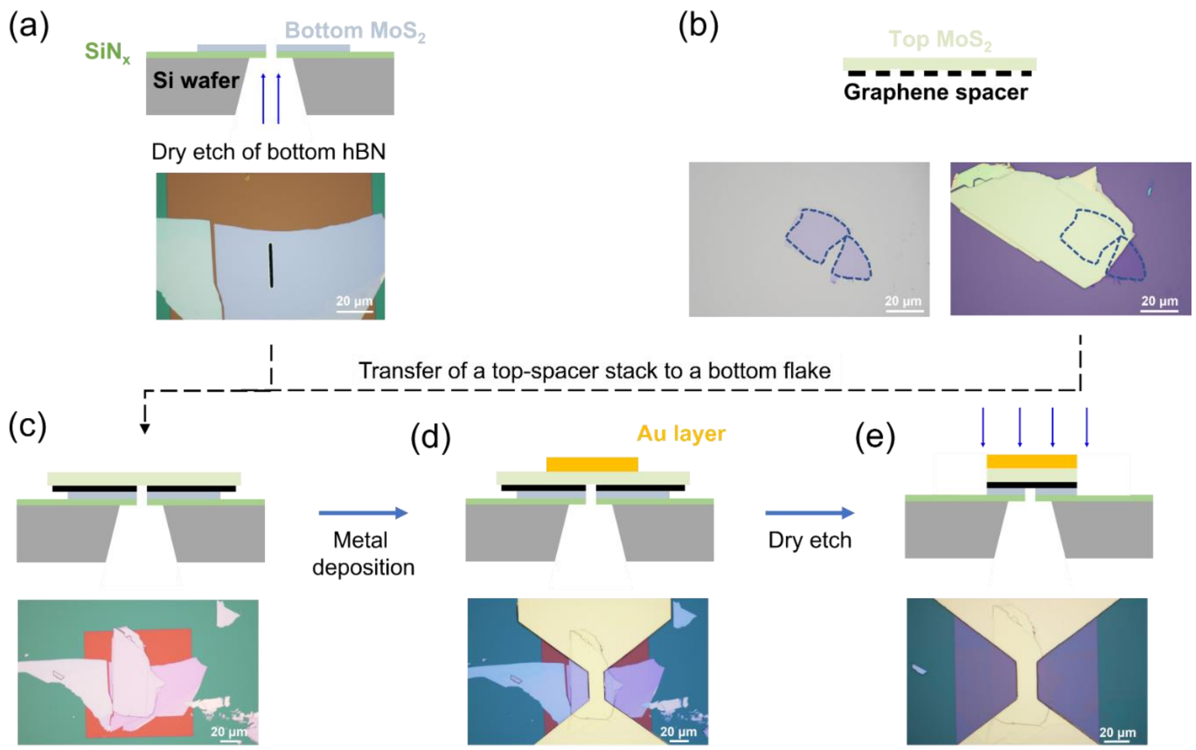

Supplementary Figure 2: Schematics and optical images with fabrication flow chart of the nanocapillary device. (a) Transfer of a MoS_2 flake to SiN_x membrane with a hole, and dry etching done from backside of the membrane. (b) Transfer of a MoS_2 flake to a patterned graphene spacer. The dotted line in optical images is a boundary of a graphene spacer. (c) Transfer of a top-spacer stack to a MoS_2 flake on SiNx membrane. (d) Au metal mask on tri-crystal stack using photolithography and metal deposition. (e) Dry etching of exposed 2D materials using RIE.

3. Conductance-voltage curves of nanochannel memristors

The instantaneous conductance G(t) is presented in Supplementary Figure 3 below. The shape of the GV curve also helps to easily distinguish the memristor type. The crossing 1 memristors (Panel A) shows higher conductance at negative polarity than at positive polarity, in contrast to the crossing 2 (Panel C) memristor, which exhibits opposite behavior. This variation in conductance states can be attributed to differences in the entrance effect of the channels, as described by Robin et al. [3], and to the surface charge of the channels that determines the type of main charge carrier or conducting ion, either an anion or a cation. The crossing 2 style occurs at low electrolyte concentrations, while crossing 1 occurs at higher electrolyte concentrations sufficient to induce charge inversion.

The non-self-crossing memristor styles, namely saturation (M2) or Wien (M4), represented in Figures B and D, show distinct GV characteristics, with both being symmetric around the y-axis. This symmetry indicates independence from the electric field's polarity. The saturation GV curve (Panel B) displays a wing-like (or M-like) structure, where the conductance jumps sharply at low starting voltages, peaking at ~0.2V, then decreases sharply as the voltage increases, reaching its minimum. Upon reversing the voltage scan and decreasing the voltage toward zero, the conductance increases again, returning to its initial value. In the case of the Wien (M4) memristor, the GV curve displays a V-like structure (Panel D), where at both positive and negative polarities, the higher the voltage, the greater the conductance becomes. This increase in conductance can be attributed to the formation of more conductive polyelectrolyte at higher voltage values than the non-conductive Bjerrum pairs present at low voltage values. In all four cases, the conductance hysteresis could be related to differences in the kinetics of ion association/dissociation or adsorption/desorption in the channels.

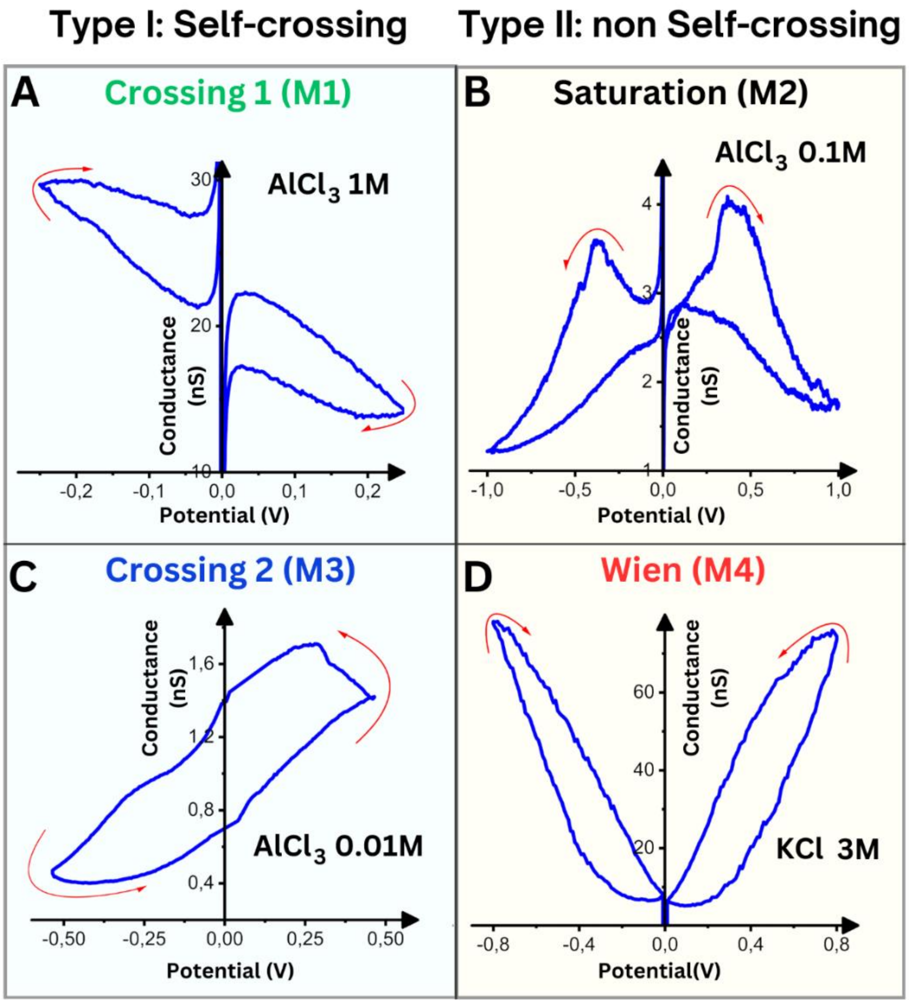

Supplementary Figure 3: Different memristor conductance styles in 2D nanochannels A-D) Typical conductance-voltage (GV) characteristic curves observed in nanochannels where either self-crossing (A-C) or non-self-crossing (B-D) memristor effects occurs depending on the experimental conditions. The instantaneous conductance was calculated by dividing the observed current over the corresponding applied voltage (G = I/V). A) Crossing 1 memristor GV curve, where the conductance increases at negative polarity (device is set) and decreases at positive polarity (device is reset). Electrical measurements were done using alternating triangular voltage of frequency 2 mHz in 2 nm device filled with 1 M $AlCl_3$ electrolyte. B) Saturation memristor GV curve, where at both positive and negative high voltages the conductance decreases, the highest conductance is observed at lower voltages (frequency 0.1 mHz, 3.5 nm thin channels, 0.1 M $AlCl_3$ electrolyte). C) Crossing 2 memristor GV curve, where the conductance increases (device is set) at positive polarity and decreases (device is reset) at negative polarity (frequency 4 mHz, 0.7 nm thin channels, 0.01 M $AlCl_3$ electrolyte). D) Wien memristor GV curve, where at both positive and negative high voltages the conductance increases due to the formation of more conductive polyelectrolyte, whereas at low voltage range the conductance is minimum due to the existence of non-conductive Bjerrum pairs (frequency 2 mHz, 2 nm thin channels, 3 M KCl electrolyte).

4. Voltage-dependence of different memristive effects

The amplitude and polarization of the applied voltage are crucial for observing the four memristive effects. The disappearance, or significant decrease of the memristive loop at low voltages and its

emergence at high voltages can be observed in Supplementary Figures 4-7. The voltage range where the hysteresis loops appear varies depending on the memristor type and the underlying mechanism. In self-crossing memristors (Supplementary Figures 4 and 6) this depends on the presence of rectification due to the asymmetrical entrance effects for either cations or anions from two ends of the channel.

In non-self-crossing memristors, the effect of voltage differs. For instance, in the Wien memristor, high voltage leads to the collective association of ions and the formation of polyelectrolyte, composed of cations and anions, which is more conductive than the Bjerrum pairs (non-conductive). This results in an increase in the device's conductance as the voltage rises (Supplementary Figure 7). There is a threshold voltage (in this device, ~250 mV) for the polyelectrolyte formation, which exhibits different conductance than the existing pools of free ions and Bjerrum pairs.

Lastly, in the case of the saturation memristor, the higher the voltage, the more pronounced the memristive effect. This could be attributed to the external polarization which is the mechanism behind the saturation effect. At low voltages, or Ohmic region, there is a constant conductivity with steady electrodiffusion. As the voltage increase and a depletion zone is formed where the electrolyte concentration tends toward zero at the channel entry, the diffusion limited current is reached (Supplementary Figure 5) [4].

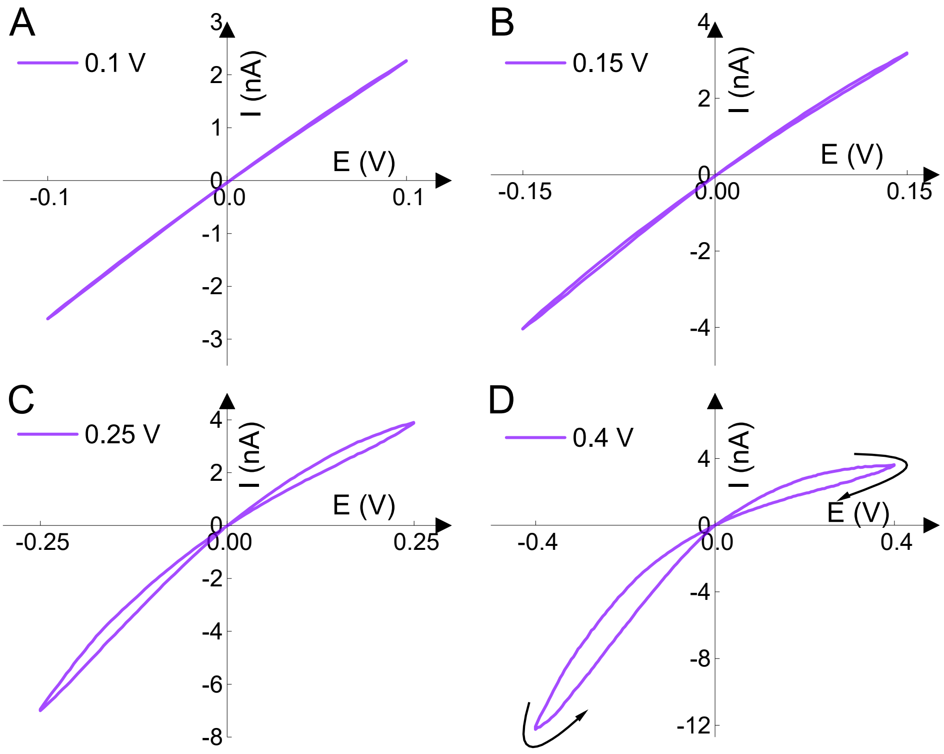

Supplementary Figure 4: Voltage effect in crossing 1 memristor. Alternating triangular voltage using different voltage ranges were applied on 2 nm hBN device using $AlCl_3$ 1M as electrolyte. The memristor effect occurs at all voltages, however, it is more evident at ≥ 250 mV.

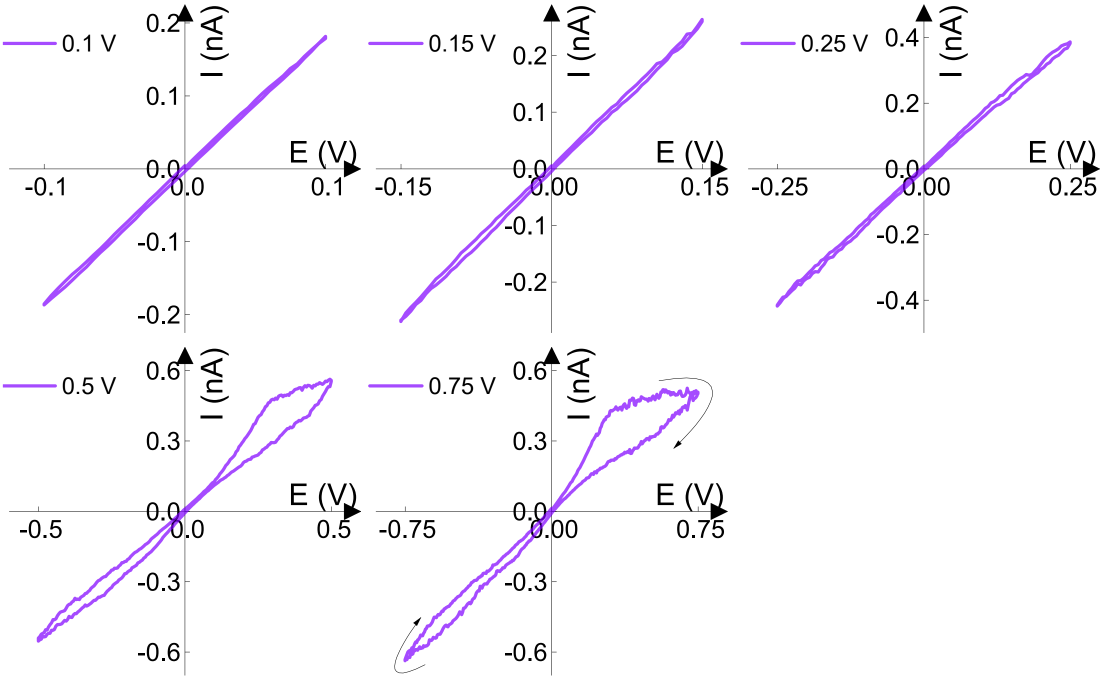

Supplementary Figure 5: Voltage effect on saturation memristor. Alternating triangular voltage using different voltage ranges were applied on 1 nm MoS_2 device using $AlCl_3$ 0.5 M as electrolyte. Voltages higher or equal to 0.5V were necessary to see the effect

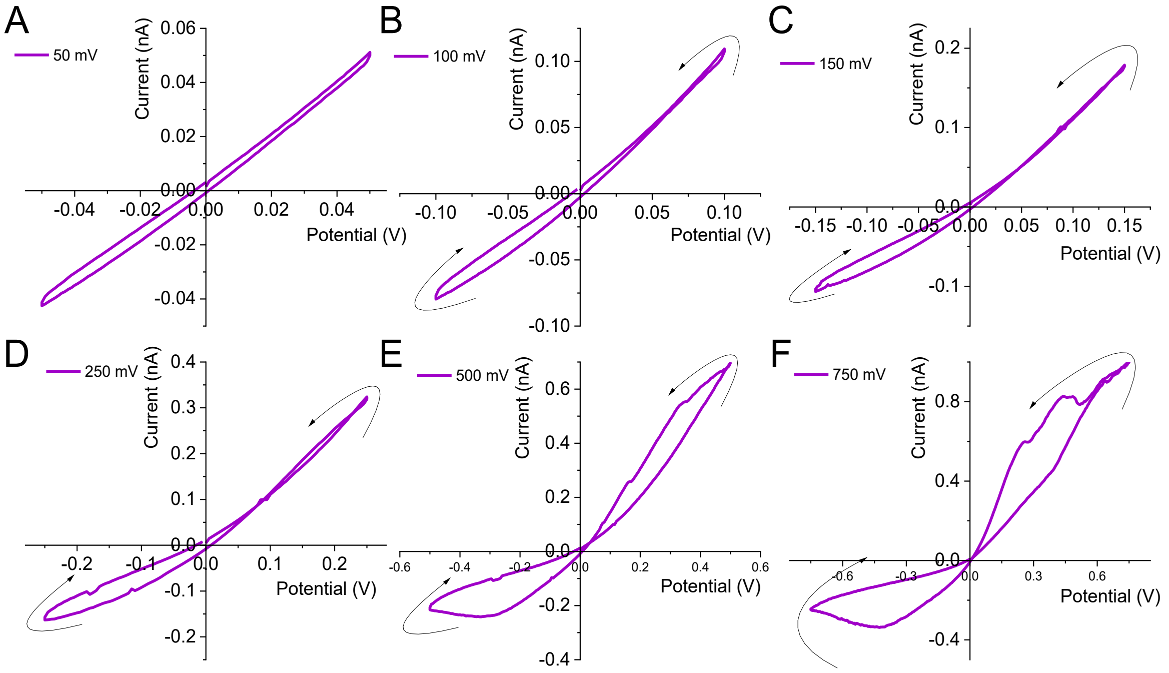

Supplementary Figure 6: Voltage effect on crossing 2 memristor. Alternating triangular voltage using different voltage ranges were applied on 0.7 nm MoS_2 device using $AlCl_3$ 0.1M as electrolyte. The memristor effect occurs at voltages as low as 100 mV.

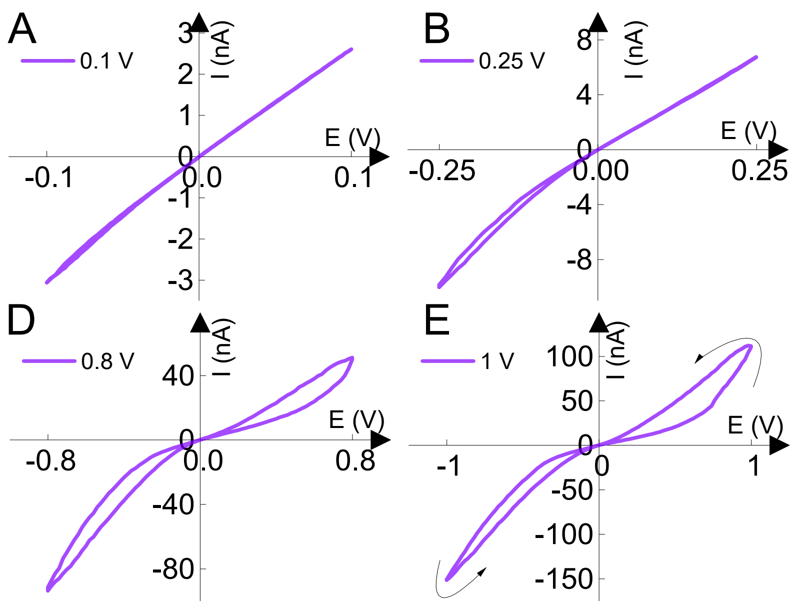

Supplementary Figure 7: Voltage effect on Wien memristor. Alternating triangular voltage using different voltage ranges were applied on 0.7 nm MoS_2 device using KCl 3M as electrolyte. Voltages higher or equal to 0.5V were necessary to see the effect on both negative and positive ends.

5. Frequency-dependence of different memristive effects

Chua et al. [5] defined the memristor as an electrical circuit element that exhibits current-voltage characteristics with pinched hysteresis. This hysteresis exhibits frequency dependence and it disappears at very high frequencies. Additionally, the IV characteristics should pass through the origin. Nanofluidic memristors demonstrate frequency dependence, as described in previous reports [3, 6]. We normalize the hysteresis area with respect to the theoretical maximum possible area that could be obtained, for various frequencies of a particular memristor. The obtained value represents the percentage memory retention at a given frequency. The maximum possible area is calculated based on the maximum and minimum conductance at positive and negative polarity and is expressed in the following equation (15):

$$A_{max,theoretical} = 0.5V_0^2x(G_{on+}\text{-}G_{off+}) + 0.5V_0^2x(G_{on-}\text{-}G_{off-}) \quad (15)$$

where G_{on+} and G_{on-} represent the highest conductance at positive and negative potentials in the GV characteristic curves, respectively, while G_{off+} and G_{off-} represent the lowest conductance. This memory retention is then plotted against the corresponding frequency. The results of different memristive effects are fitted according to the equation (16) derived in our previous report [3].

$$A^*(\omega) \cong K\frac{\omega\tau_m}{1+\omega^2\tau_m^2} + c \quad (16)$$

Where, A* is the normalized area, ω is the angular frequency, K is a constant used as a fitting parameter, τ_m is the memory timescale, c is a constant that represents the residual memory that occurs at high frequencies in part due to emerging capacitive currents. The four memristive patterns follow the same trend where they fit the equation (16), however, the characteristic τ_{max} and $percentage\ memory\ retention_{max}$ differ depending on the memristor style (Supplementary Figures 8). The saturation memristor consistently showed the lowest τ_{max} and the highest maximum retention, signifying a strong memristive effect. This could be attributed to the slow dynamics of

establishing a concentration gradient at the entry of the nanochannels. Conversely, the Wien effect memristor showed the highest τ_{max}.

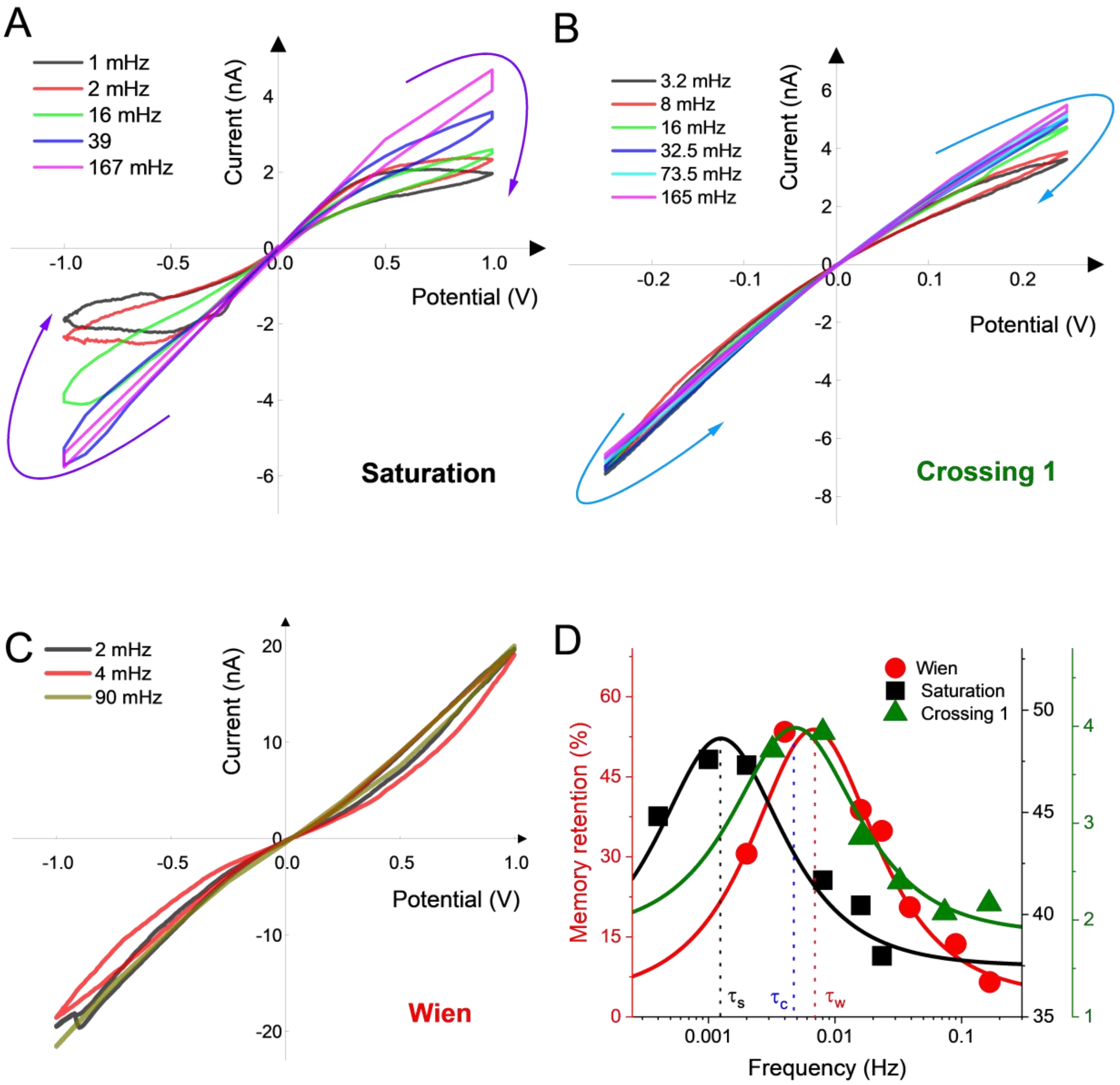

Supplementary Figure 8: Frequency-dependence of various memristive effects. Current-voltage characteristics of A) 0.7 nm MoS_2 device showing saturation-type memory (electrolyte, 0.5M $AlCl_3$), and B) 2 nm hBN device showing a crossing 1 memristor (electrolyte, 1M $AlCl_3$) and C) 0.7 nm MoS2 device showing Wien memristor (electrolyte, 1M $Al_2(SO_4)_3$) at various alternating voltage frequencies. D) Comparison of the memory retention for Wien (Red circles), crossing 1 (Green triangles) and saturation-style (Black squares) memristors as a function of the applied voltage frequency. The memory timescale (τ) can be extracted from experimental data by identifying the frequency at which the loop is the largest. The saturation curve has a higher memory constant than the crossing 1 and the Wien curve (inversely proportional to the frequency).

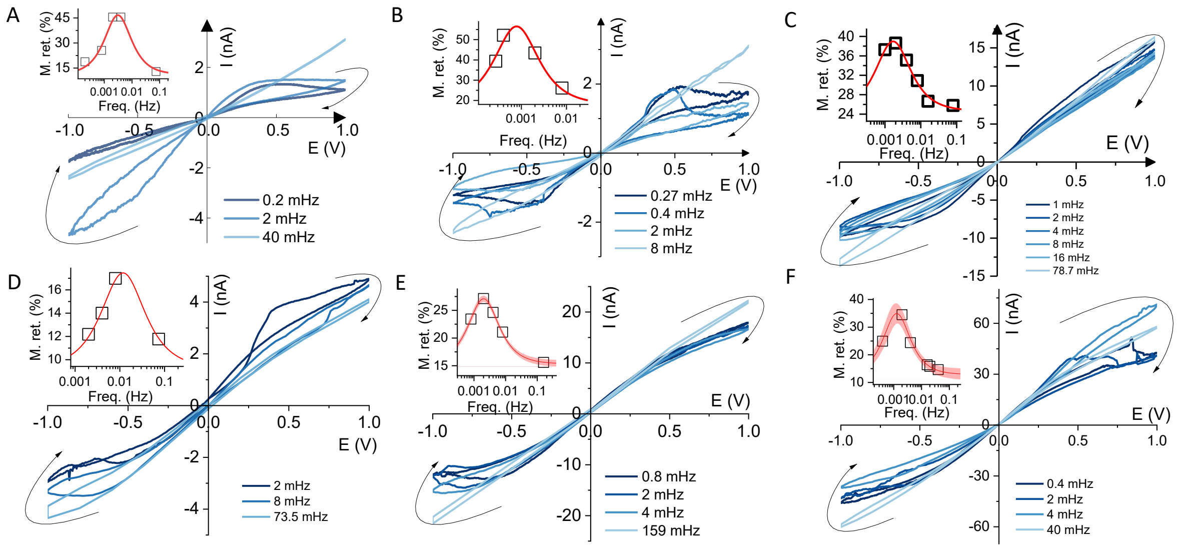

Supplementary Figure 9: Frequency effect in saturation memristors. Current-voltage characteristics and percentage of memory retention (insets) curves upon application of $\pm 1\ V$ alternating triangular voltage of different frequencies using devices with variable thicknesses and electrolyte ($AlCl_3$) concentrations A) h=2 nm, hBN, C=0.1M, B) h=3.8 nm, hBN, C=0.1M, C) h=0.7 nm, MoS_2, C=1M, D) h=20 nm, MoS_2, C=0.001M, E) h=20 nm, MoS_2, C=0.01M, F) h=20 nm, MoS_2, C=0.1M.

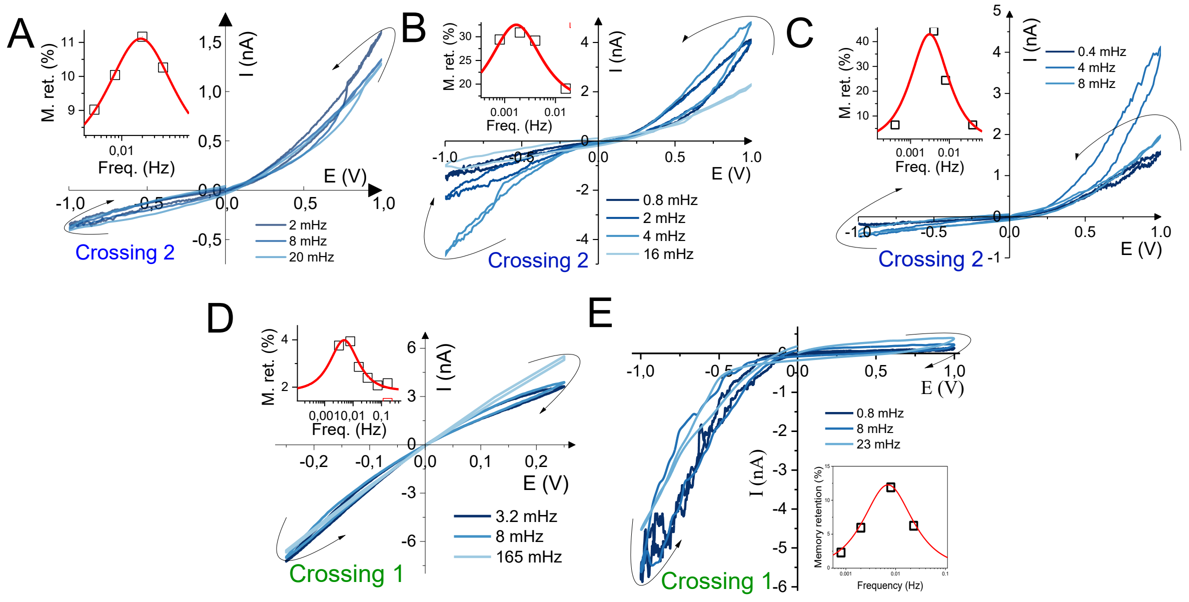

Supplementary Figure 10: Frequency effect in crossing (1 and 2). Current-voltage characteristic and percentage of memory retention (insets) curves upon application of ±1 V alternating triangular voltage of different frequencies using devices with variable thickness and electrolyte A) Crossing 2 (h = 2 nm, hBN, 0.01M $AlCl_3$), B) Crossing 2 (h = 3.8 nm, hBN,1M $Al_2(SO4)_3$), C) Crossing 2 (h = 3.8 nm, hBN, 0.1M $Al_2(SO4)_3$), D) Crossing 1 (h = 2 nm, hBN, 1M $AlCl_3$), E) Crossing 1 (h = 1.4 nm, hBN, 4M $MnCl_2$).

6. Surface charge inversion

Relating to the discussion in the main article about memory type crossing 1, we will delve deeper into the analysis of the surface charge inversion analysis. The main charge carriers

inside nanochannels can be anions, cations, or both, each contributing in various proportions. To determine the primary contributors, we conducted two types of experiments.

The first experiment is diffusion, where we examine the rectification using a concentration difference of electrolyte on either side of the nanochannels. By using a gradient of 100, the ion concentration on the lower concentration side becomes negligible compared to the higher concentration side, minimizing the contribution of its ions, whether cations or anions. When the potential and concentration gradients align, as observed at positive polarity, both forces drive the cation through the nanochannel while counteracting the anion. In this scenario, the cation is the major contributor to the observed current, as the anion's concentration in the opposite compartment is too low to significantly affect the current value. Conversely, at negative polarity, when the concentration gradient opposes the potential gradient, the cation is impeded by the concentration gradient, while the anion is facilitated by it, making the anion the primary contributor to the current in this configuration

This phenomenon is illustrated in Supplementary Figure 11, where at low sodium chloride (NaCl) concentrations (0.1 - 0.001M), the positive current significantly exceeds the negative current, indicating that the sodium cation is the main ion transport contributor. These observations could be explained by the negative surface-controlled regime of the hBN device, where the walls hinder the entry of Cl^- (Figure 3 in the main). However, at high NaCl concentrations (6M to 0.06M), the situation reverses, with the negative current much higher than the positive current. This suggests that the chloride ion contributes more to the current, a situation that could only occur if charge inversion at the hBN walls prevented additional Na^+ entry (Figure 3c). Additionally, the asymmetric nature of the channels, arising from disparities in crystal charge distribution between devices and variations in entrance resistance, should be acknowledged. Consequently, the pathways for cation and anion entry into the channel may differ. The mobility of ions within nanochannels varies significantly, influenced by factors like the hydrated and ionic diameter of the ions [7, 8]. The reduced mobility and lower conductance of bivalent cations can limit their dominance at lower concentrations, making the actual dynamics more complex than this simplified approximation suggests.

This methodology to study surface charge inversion (SCI) using asymmetric solution concentrations (concentration gradient) was reported previously. For example, He et al. [9] demonstrated charge inversion by comparing monovalent (K^+ and Cl^-) and bivalent (Ca^{2+} and Cl^-) solutions where they observed SCI only using $CaCl_2$ solution. Additionally, Li et al [10] predicted using simulations the SCI to occur in asymmetric conditions when bivalent ions rather than monovalent ions are used. They used a methodology similar to ours by keeping a fixed concentration gradient and varying the concentration of electrolyte on both sides of a nanochannels (Figure 3). Li et al [11] reported various concentrations of electrolyte using fixed gradient on PET etched nanopore, and correlated the rectification factor (RF) variation with SCI. They observed SCI only in bi and trivalent salts and not using monovalent KCl solution. We observed similar phenomena as described by Li et al. [10] with our methodology (fixing one electrolyte gradient 100 and varying the cis concentration), though the concentration range for charge inversion was higher (M vs mM), likely due to our specific system's 2D confinement in ultrathin slits. This shift in the concentration necessary for SCI towards a higher range between bulk and confined systems was also noted by Morikawa et al. [12] and attributed to a more highly charged surface in extended nanospace. Additionally, Li et al [11]

observed SCI in molar and higher millimolar ranges for bivalent and trivalent ions, respectively, in PET nanopores.

The second experiment involves simply the observation of the change of the RF with increasing the concentration of the electrolyte.

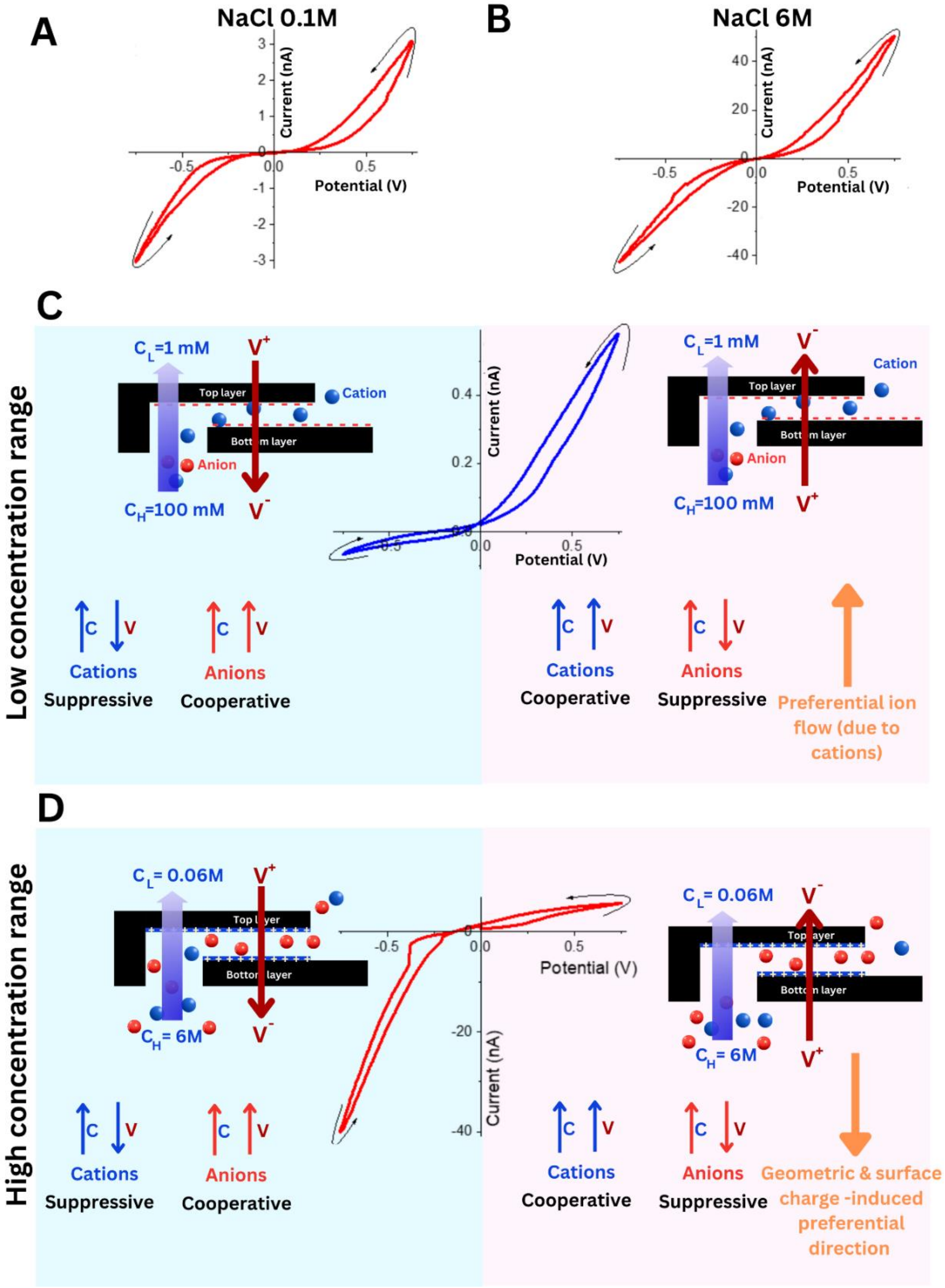

Supplementary Figure 11: Diffusion gradient experiments at high and low salt concentrations. equimolar current-voltage characteristics of NaCl at A) relatively low (0.1M) and B) high (6M) concentrations. C-D) The diffusion experiment was conducted by establishing a concentration gradient

of 100 across the nanochannel: C) 100 mM: 1 mM and in D) 6M : 0.06M, with the higher concentration originating from the hole side. The direction of the voltage polarization, where positive voltage was applied, was the same as the concentration gradient while it was opposite to the concentration gradient when negative voltage was applied. C) At a low NaCl concentration with a gradient, the positive current significantly exceeded the negative current. This indicates that the predominant factor of ion transport is the cation (Na^{+}) as their individual concentration and electric gradient cooperate (those of anions suppress each other) (light red region). In the light blue region the anion's concentration and electric gradient cooperate while those of cations suppress. D) Conversely, at a high NaCl concentration gradient, the situation was reversed, where the negative current (light blue region) exceeds the positive one (light red region) signifying that the predominant factor of ion transport is the anion (Cl^{-}) as their individual concentration and electric gradient cooperate (those of cations suppress each other). This suggests a charge inversion, thereby rendering the anion (Cl^{-}) the principal agent.

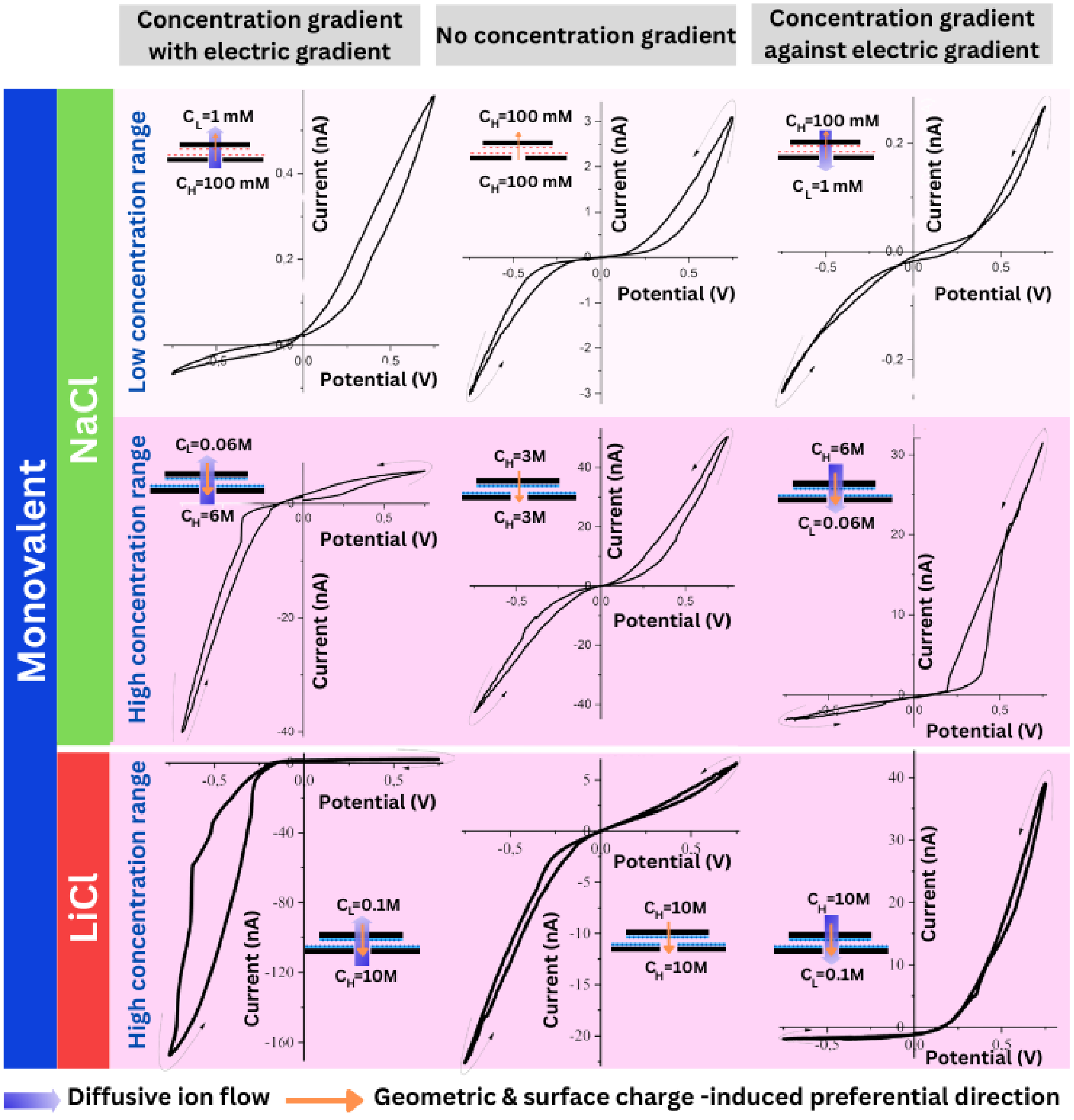

Supplementary Figure 12: Diffusion gradient experiments at high and low salt concentrations for monovalent salts. The rectification factor (RF(+/-))—the ratio of positive to negative current—was

evaluated under equimolar conditions and with a salt concentration gradient. For low concentration gradients of monovalent salts (NaCl, LiCl), RF(+/-) increases when the gradient is applied from the hole to the device compared to equimolar concentration, indicating a preferential ion flow in this direction without surface charge inversion. In contrast, at high concentrations of monovalent salts (NaCl, LiCl), RF(+/-) increases when the gradient is reversed (device to hole), reflecting surface charge inversion with anions as the dominant charge carriers. Experiments were performed on an hBN device with a channel height of 1.4 nm.

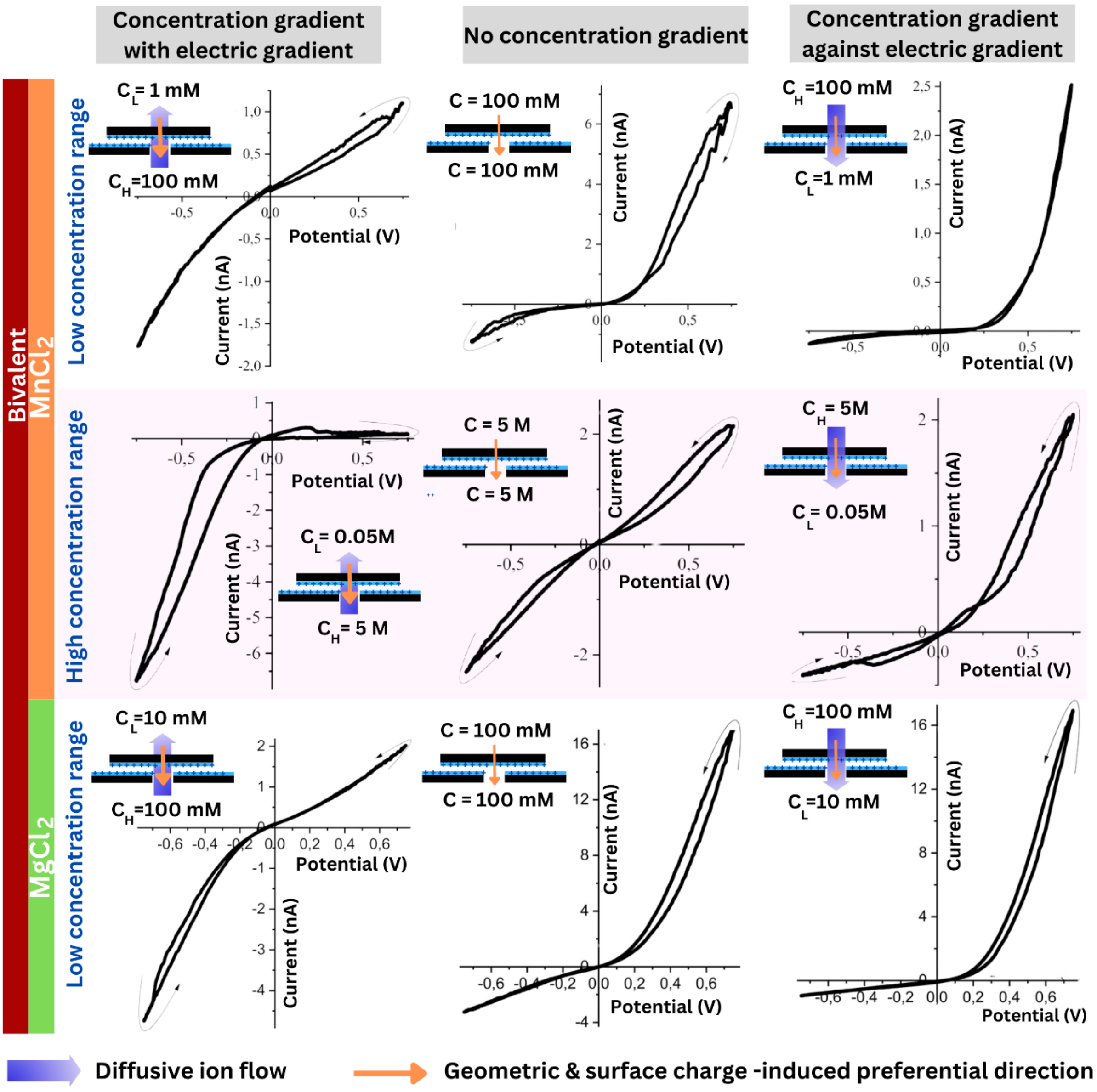

Supplementary Figure 13: Diffusion gradient experiments at high and low salt concentrations for bivalent salts. The rectification factor (RF(+/-))—the ratio of positive to negative current—was evaluated under equimolar conditions and with a salt concentration gradient. For all concentrations of bivalent salts ($MnCl_2$, $MgCl_2$) RF(+/-) increases compared to equimolar concentration when the gradient is from device to hole, reflecting surface charge inversion with anions as the dominant charge carriers. Experiments were performed on an hBN device with a channel height of 1.4 nm.

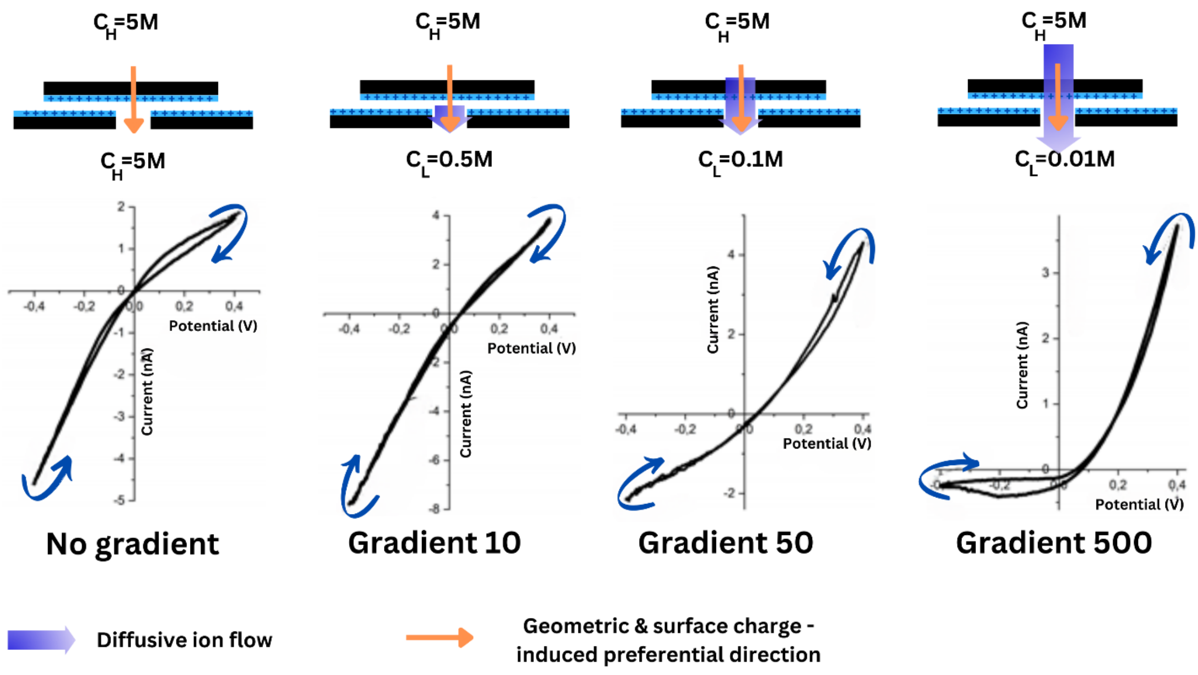

Supplementary Figure 14: Effect of the concentration gradient on the current rectification. The rectification factor (RF(+/-)) — the ratio of positive to negative current — reverses as the concentration gradient of $MgCl_2$ increases from the device side to the hole side. Experiments were performed on an hBN device with a channel height of 1.4 nm using $MgCl_2$ as the electrolyte. The $MgCl_2$ concentrations on the hole and device sides are shown in the schematic above each IV curve. At the high concentrations used, surface charge inversion occurs.

7. Change of memristive style by concentration or pH

With simultaneous existence of various mechanisms, varying one parameter would result in a change in the loop style by either enhancing the presence of one memristor mechanism or suppressing a concurrent mechanism. The following experiment aims to facilitate switching between different memristor styles by varying two parameters: concentration and pH.

The effect of concentration is thoroughly described in the main text with the 3D plot in figure 2. However, it is further emphasized here through normalization with respect to the maximum current of each curve (Normalized I = $I/|I_{max}|$). At low concentrations, the crossing 2 effect predominates in hBN and MoS_2 devices. However, at high concentrations, the Wien effect predominates, especially in MoS_2 devices with monovalent ions. High concentrations are crucial for allowing both cations and anions to enter the channel, thus forming Bjerrum pairs and polyelectrolytes in the Wien memristor. Conversely, low concentrations are essential for the crossing 2 memristor to occur, as the surface charge of the channels should not be shielded by a high number of counter ions entering the channel when the electrolyte concentration increases.

We observed change in RF, relative variation in positive and negative conductances (G_+/G_-), with increasing the concentrations of electrolytes (Supplementary Figure 16). This could coincide with the type of loop change from Crossing 2 to Crossing 1, that occurs mainly when using a multivalent cation with hBN nanochannels. However, even when using monovalent cation salts where Wien effect occurs predominantly (Figure 2), the effect of RF change remains pronounced (Supplementary Figure 16). This change in RF with monovalent cations in a memristor showing Wien effect is noticeable when

using lithium cation (Figure 3 in main), which has smaller ionic diameter and larger hydrated diameter than other monovalent cations such as K^+ and Na^+ (Supplementary Figure 16) So, monovalent ions also can experience charge inversion at very high concentrations revealed by the change of RF without having the crossing 1 style as the final output. This supports the existence of simultaneous memristance mechanisms. This effect of variation of RF is also seen in MoS_2 nanochannels, where the crossing 1 at high concentration was never seen (Supplementary Figure 17). This signify that the absence of Crossing 1 at high salt concentration in MoS_2 nanochannels is mostly due to the predominance of Wien effect rather than the absence of ion adsorption and SCI.

The effect of pH is particularly significant in examining the influence of surface charge on the observed memristive behaviour. The "crossing 2" memristor hysteresis mechanism arises from the differing kinetics of adsorption/desorption of counterions (e.g., cations for a negatively charged surface) on the channel surface. In contrast, the Wien effect is governed by the differing kinetics of association/dissociation of free ions into Bjerrum pairs. In this study, a device was initially tested under conditions favouring a surface-charge-driven memristive mechanism (crossing 2) using a neutral KCl solution (pH 5.5, 10 mM concentration). Upon adding HCl to adjust the pH to 3, a transition from the crossing 2 mechanism to the Wien effect was observed. The concentration of HCl was negligible relative to KCl, indicating that the memristive behaviour remained primarily governed by the KCl electrolyte. These observations confirm two key points: (1) surface charge plays a critical role in enabling the crossing 2 mechanism, and (2) the simultaneous presence of both mechanisms allows for one effect to dominate when the other diminishes.

The nanochannel system is in a balanced state between at least two memristor effects, and changing the surface or electrolyte parameters can tilt the balance towards either memristive style. The balance between the surface and the solution effects could be seen when changing the anion for the same cation electrolyte. Wien memristor occurred at high concentrations of sulfate, while crossing 1 occurs at high concentrations of chloride salts in hBN devices. This reflects the effect of the anion on the memristor style, where the sulfate, due to its bivalent charge, can interact more with the bi and trivalent cations thus enhancing the appearance of Wien memristor. The bivalent electrolytes of chloride salts did not show crossing 1 mechanism in MoS_2 devices, which are known to possess slightly higher negative surface charge than the hBN. This could explain the enhancement of the Wien effect and the masking of the “self-crossing” effects.

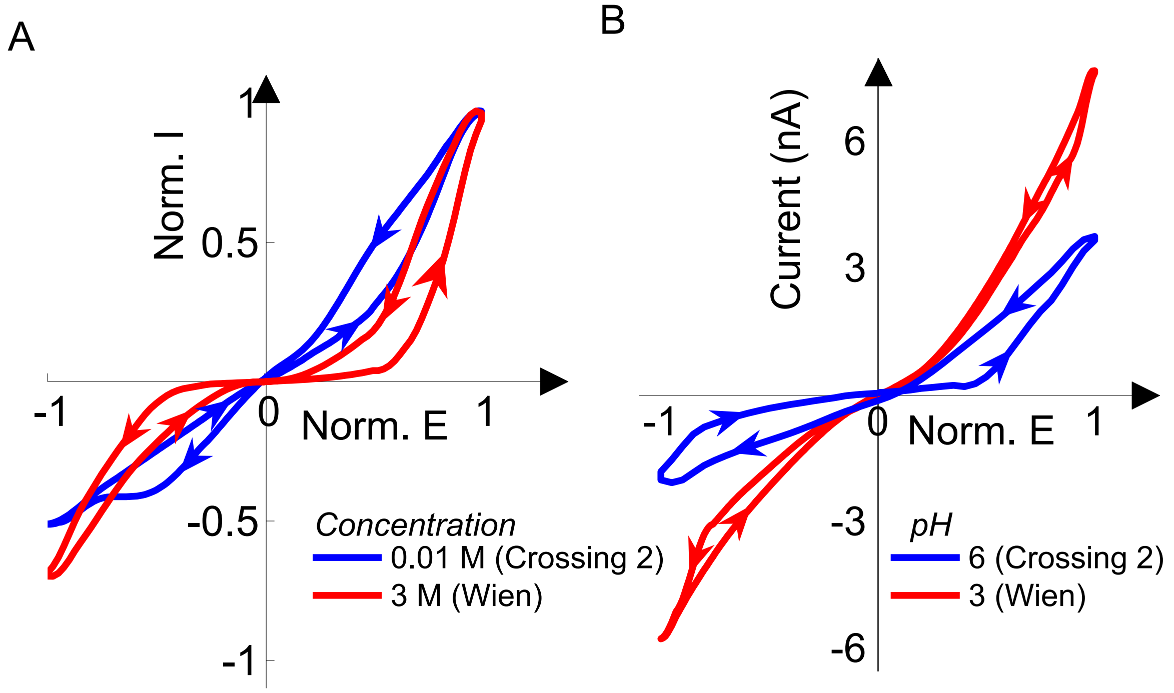

Supplementary Figure 15: Inversion of Memristor loop style. Current-voltage characteristics of nanochannels (height, 7 nm with hBN top and graphene bottom) using KCl as electrolyte showing the inversion between the Wien and the crossing 2 memristor styles depending on the A) concentration of the electrolyte (KCl, 10 mM and 3 M) and B) the pH of the solution (KCl 0.1M, acidic pH 3 and neutral pH 6). The red color curve represents Wien effect while the blue color represents adsorption effect.

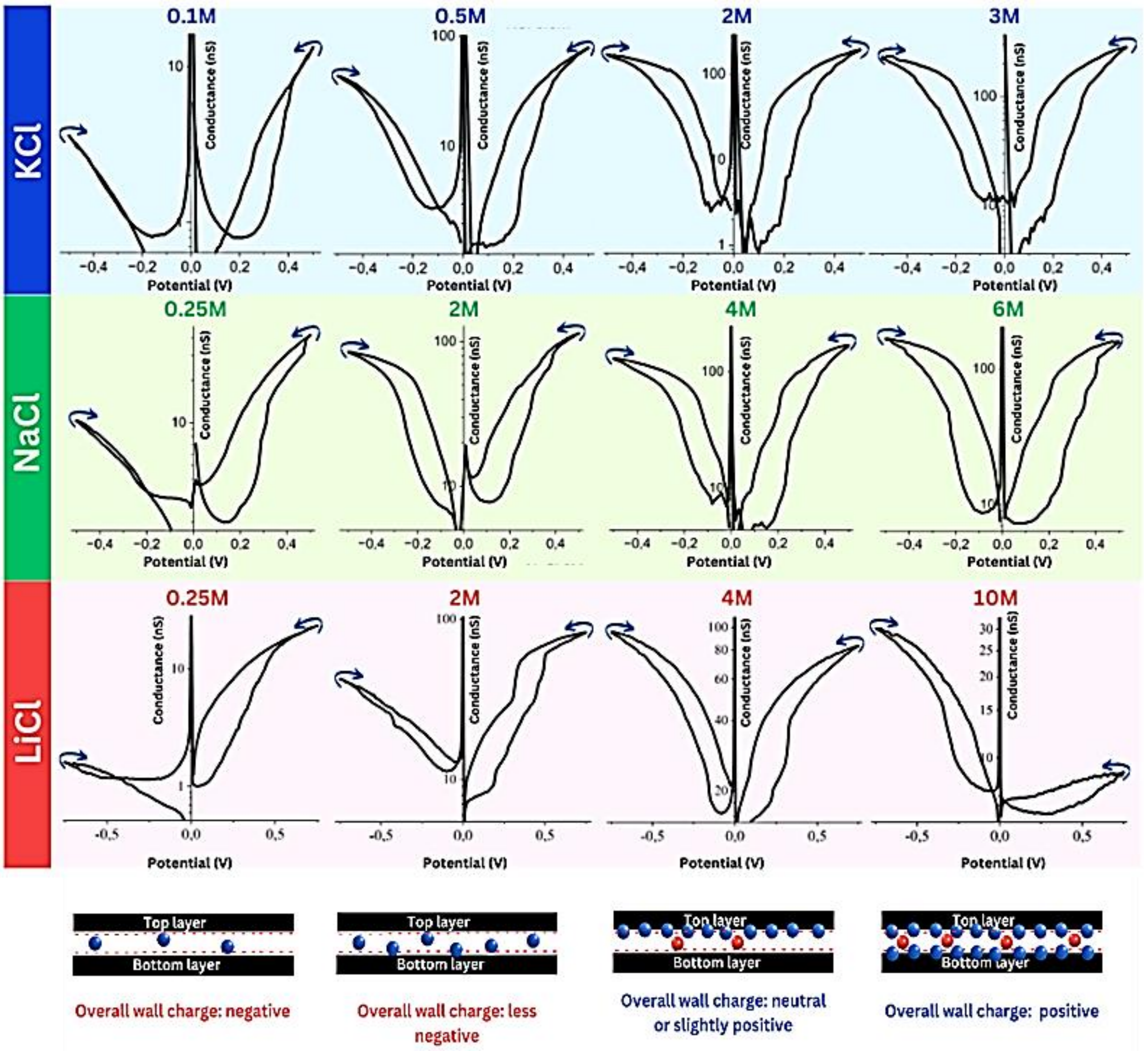

Supplementary Figure 16: Effect of increasing electrolyte's concentration on the current rectification in hBN device. Conductance-voltage (GV) characteristics of KCl, NaCl, and LiCl electrolytes at equimolar concentrations in hBN channels (h = 1.3 nm). The GV curves are shown for increasing salt concentrations, as indicated above each plot. All systems exhibited the Wien effect, with the ratio of positive to negative conductance (RF) varying as concentration increased. This behaviour suggests simultaneous charge inversion, likely due to changes in the relative proportions of cations and anions in the electrolyte. The highest concentration tested corresponds to the maximum solubility of each salt. Among the salts, LiCl showed the greatest RF variation followed by NaCl, and then KCl. This variation in RF suggests an accumulation of cations (blue circles), which blocks further cation entry into the channel, making anions the primary carriers of current transport.

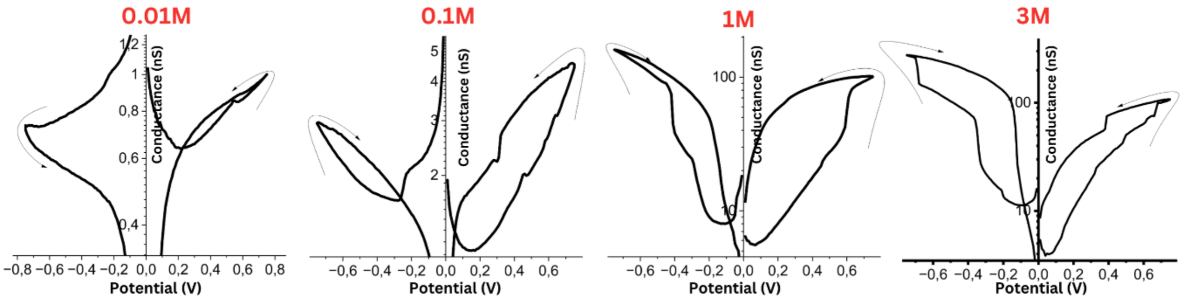

Supplementary Figure 17: Effect of increasing LiCl concentration on the current rectification in MoS_2 device. Conductance-voltage (GV) characteristics of LiCl electrolyte at equimolar concentrations, ranging from 0.01 M to 3 M, using MoS_2 channels (h = 0.7 nm). The ratio of positive to negative conductance changes with increasing concentration, suggesting simultaneous charge inversion, due to variations in the relative proportions of cations and anions in the electrolyte. LiCl 0.01M shows M3 (crossing effect) while for concentrations higher than 0.1M, all the observed memristive effects were Wien effect (M4).

8. Memristor variation with channel height and electrolyte concentration:

The results in this section provide a visual summary map of how the height of the channels and the concentration of KCl electrolyte affect the variation of the memristive effect. Notably, as the height increases (beyond 10 nm), the crossing 2 memristive effect becomes the dominant effect, even at high KCl concentrations (1M and 3M) albeit with small hysteresis loop (Supplementary Figure 18). However, as the electrolyte concentration increases, the Wien effect becomes predominant in most instances, except in thicker channels where the crossing 2 memristive effect remains dominant. The increase in electrolyte concentration enhances the Wien effect by raising the number of cations and anions in the nanochannels, thereby increasing the number of Bjerrum pairs. It also reduces the crossing 2 adsorption due to the screening of surface charge by the counterions. Thus, the Wien effect becomes predominant and emerges as the overall effect. However, the formation of Bjerrum pairs is inversely proportional to the height of the channel, as discussed by Robin et al [6]. Therefore, in thicker channels, even at high concentration, the Wien effect does not occur, but a small crossing 2 effect persists, resulting in a very slight overall crossing 2 hysteresis (Supplementary Figure 19).

Similarly, in the case of $AlCl_3$ electrolyte, the lower height (more confined nanochannels) show better loop with saturation (M2) memritive effect (Supplementary Figure 21). This effect decreases with the height of the nanochannel but it can persist to heights upto 20 nm (vs 10 nm for KCl Wien effect) (Supplementary Figure 21). Using very thick nanochannels the hysteresis area decreases a lot and there is a tendency toward obtaining an ohmic behaviour. This reflects in both cases, using KCl or $AlCl_3$, the importance of 2D confinement in obtaining memristive effect.

The results in Supplementary Figure 20 provide summary of how channel height and electrolyte concentration affect the memristive effect in nanochannels with MoS_2 and hBN walls. The electrolyte tested here contains the trivalent Al^{3+} cation. Notably, both in MoS_2 and hBN devices, the saturation style (indicated by black symbols) occurs at low concentrations (≤0.1M) across all channel heights and other types of loops appear at high concentrations for all tested heights. This phenomenon could be attributed to the inherent mechanism of the saturation memristive style, i.e., concentration polarization.

In MoS_2 devices, Wien effect occurs in low channel heights (<10 nm) and high salt concentrations (>1M). At higher heights, crossing 2 effect exist but with very small loop areas. This could be explained

by the decrease in selectivity of the nanochannels which does not permit the conditions for external polarization to occur and thus the adsorption/desorption occurs. It is important to note that as the concentration and height of the channel increase, selectivity decreases, leading to a diminished saturation effect and to the decrease of the area of the loop (Supplementary Figure 21).

In hBN devices, the emergence of the crossing-1 mechanism (green down triangles) occurs at low heights and intermediate to high concentrations. This phenomenon is due to the surface charge inversion mechanism that is pronounced with multivalent ions (here Al^{3+}), where the negative surface charge of the channel turns positive through the adsorption of Al^{3+} ions, and the hysteresis mechanism results from the difference in kinetic mechanisms of adsorption and desorption. The conditions for this memristor are – thin channels for the selectivity and rectification. Wien effect occured at heights > 3nm which involves both cations and anions. In even thicker channels, the memristive effect disappears and tend more toward small crossing 2 effect.

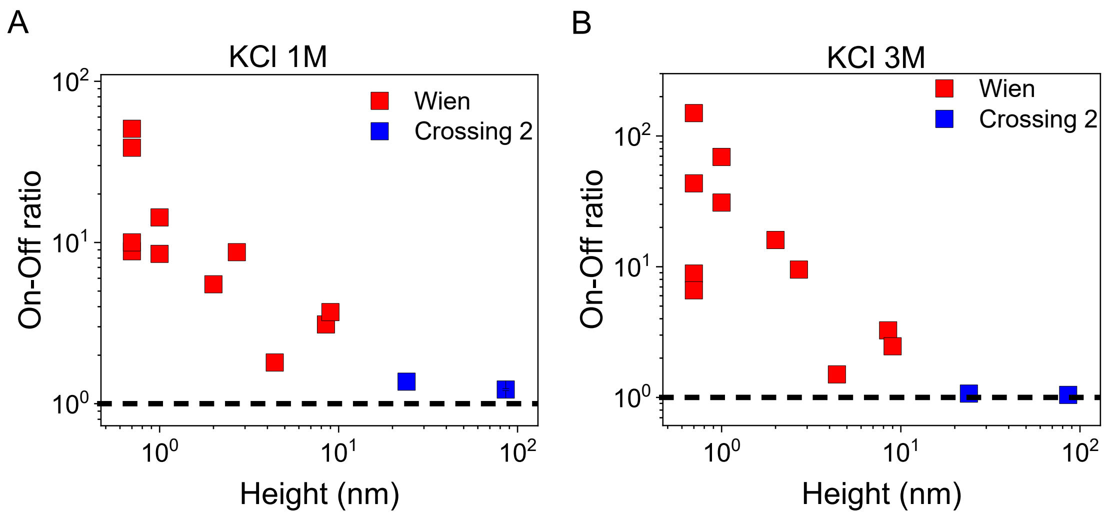

Supplementary Figure 18. Memristor dependence on channel height in KCl electrolyte. The On-off conductance ratio of MoS_2 nanochannels of different thicknesses using A) 1 M KCl, and B) 3M KCl as electrolyte. The different observed memristor effects are represented by different colors (Wien: red, crossing 2: blue). Alternating voltage range is between $\pm 1\ V$.

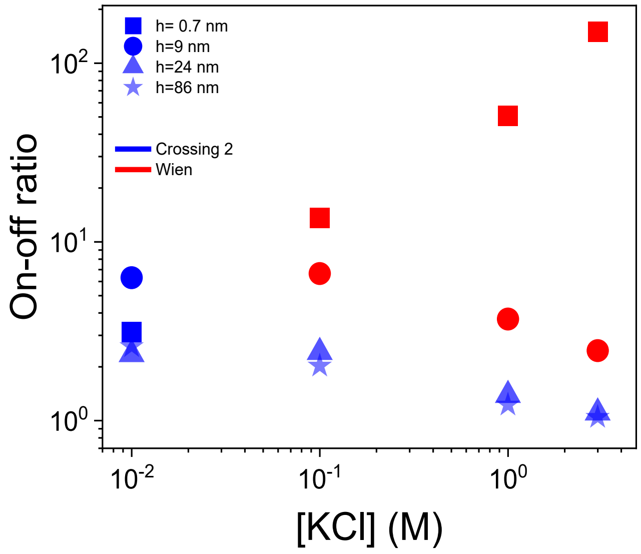

Supplementary Figure 19: Memristor dependence on KCl electrolyte concentration. The On-off conductance ratio of MoS_2 nanochannels of different thicknesses using KCl as electrolyte (square: 0.7 nm, circle: 9 nm, triangle: 24 nm, and star: 86 nm). The different observed Memristor effects are represented by different colors (Wien: red, crossing 2: blue). Alternating voltage range is between $\pm 1\ V$.

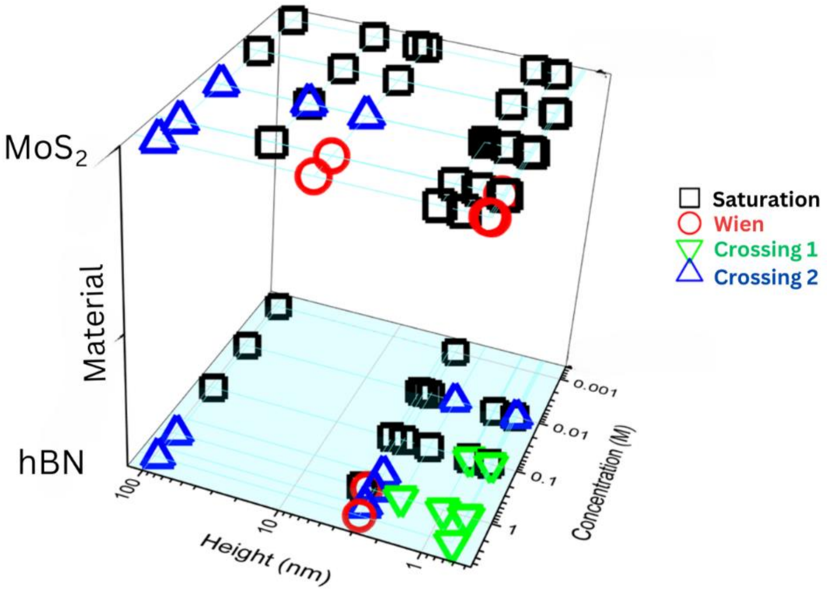

Supplementary Figure 20: Memristor dependence on $AlCl_3$ electrolyte concentration, height of the nanochannel and the material of the nanochannel. The different observed memristive effects (Saturation, Wien, Crossing 1 and crossing 2) are represented by different colors and shapes. Alternating voltage range is between $\pm 1\ V$.

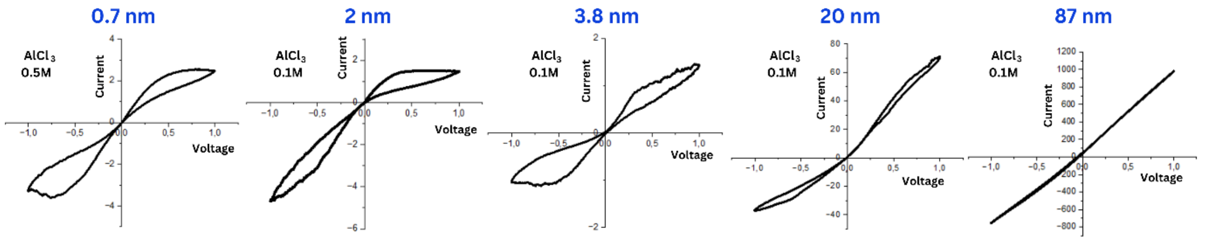

Supplementary Figure 21: Memristor dependence on nanochannel height using $AlCl_3$ electrolyte. Current-voltage characteristics showing the decrease of memristive loop area with channel height. Measurements were performed under an alternating voltage sweep, $\pm 1\ V$, the salt concentration and 2D channel material are indicated over each figure panel.

9. Effect of electrolyte salt type

The study explores the influence of the electrolyte salt's cation, where the effects of mono- and bivalent cations of chloride salts on memristive behaviour is tested, utilizing hBN devices at intermediate concentrations. The aim is to examine the salts' affinity for inducing charge inversion and the emergence of saturation memory. It's important to remember that multiple mechanisms can coexist simultaneously, but the predominant mechanism determines the overall observed effect. The experiments, which maintained Cl^- as the anion at 1M concentration, revealed that all monovalent salts exhibited a Wien effect memristor (Supplementary Figure 22). However, bivalent salts displayed varied behaviours: some salts (e.g., Ca^{2+}) predominantly showed the Wien effect, while others like Mg^{2+} exhibited a double crossing away from the origin (Supplementary Figure 22). This indicates the presence of two coexisting effects — saturation at high voltage and crossing at low voltage — whose expression varies with the voltage. Additionally, Mn^{2+} electrolyte consistently demonstrated a saturation effect (Supplementary Figure 23). These variations could be attributed to differences in selectivity between the cation and the anion, coupled with cation adsorption to the surface, which makes the conditions of obtaining ion concentration polarization regime more probable. This could be coupled with the fact of existence of agglomerations of Mn salts (complex dihalogenoaqueous forms) as discussed in the main document. Similarly, trivalent cations, such as Al^{3+}, demonstrate a pronounced saturation effect, attributed to their high selectivity versus chloride anions and their adsorption effect coupled with the existence of polymeric Aluminum complexes.

The overall observed effect results from interactions between the cation, solvent molecules, surrounding anions, and the channel wall's surface charges. Hence, investigating the impact of the electrolyte anion type was critical. All cation valencies (including Mg^{2+}, Mn^{2+} and Al^{3+}) exhibited a Wien effect with sulfate at intermediate 1M concentrations, contrasting with observations from chloride salts, where bivalent and trivalent cations showed a saturation effect (Supplementary Figure 24). This indicates the diminishing influence of surface charge inversion and the selectivity of bivalent and trivalent cation electrolytes. A simple switch between anions of the same cation and concentration highlights the anion's effect. For instance, changing the anion in $AlCl_3$ from chloride to sulfate altered the memory loop style from saturation to Wien or Crossing 2, depending on the concentration used. This emphasizes the significance of interactions among the cation, anion, and channel surface charge in determining the apparent memristive effect.

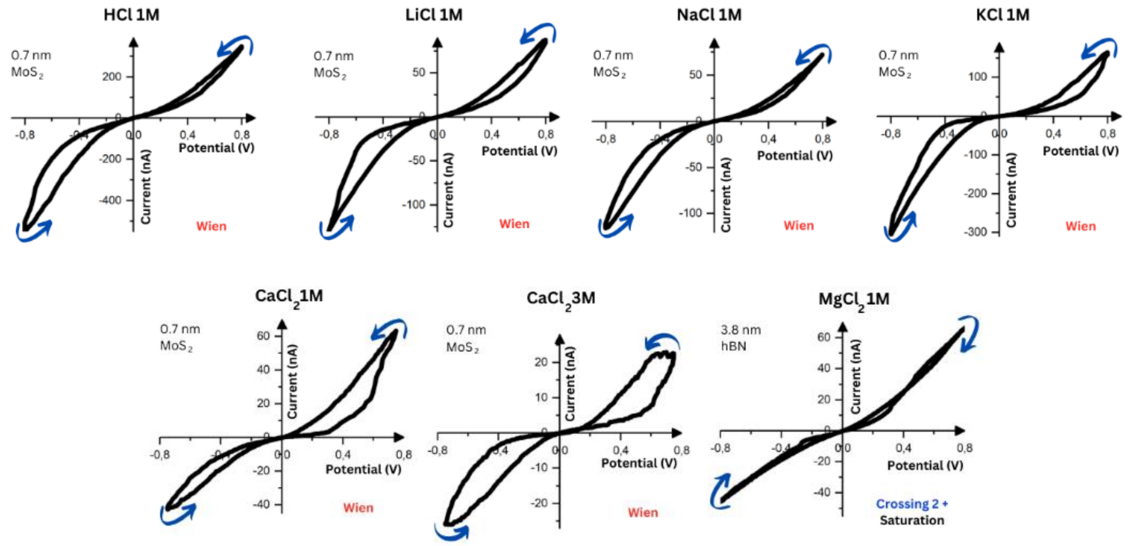

Supplementary Figure 22: Memristor effect using chloride salts of different cations. Current-voltage characteristics of nanochannels filled with different 1 M electrolytes composed of different cations (H^+, Li^+, Na^+, K^+, Ca^{2+}, Mg^{2+}). The heights of the devices, the salt and the frequency is written on each IV curve. Wien effect memristor is observed in all the above salts with the exception of $MgCl_2$ that shows mixed effect with double crossings (at the given concentration).

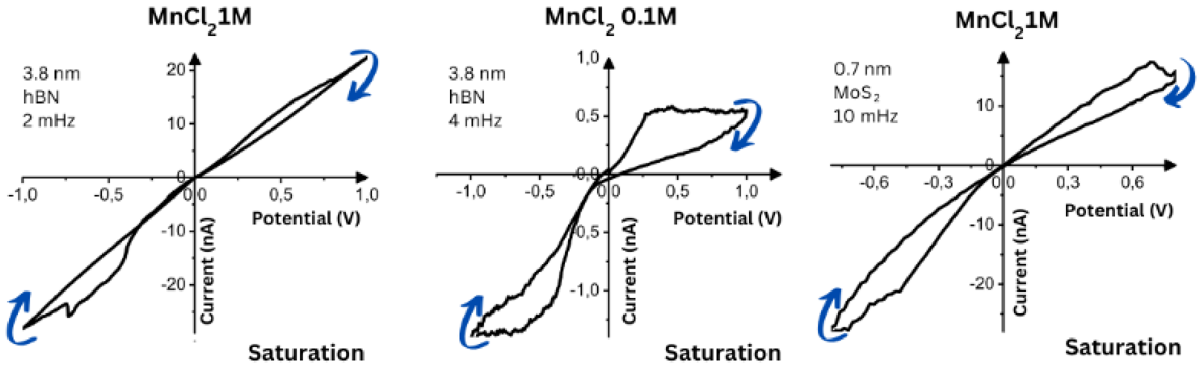

Supplementary Figure 23: Saturation memristor IV curves using $MnCl_2$ electrolyte. Current-voltage characteristics of nanochannels filled with $MnCl_2$ electrolytes under alternative triangular voltage. The heights of the devices are 3.8 nm in A-B and 0.7 nm in C. The concentration of $MnCl_2$ is 1M in A, C and 0.1 M in B

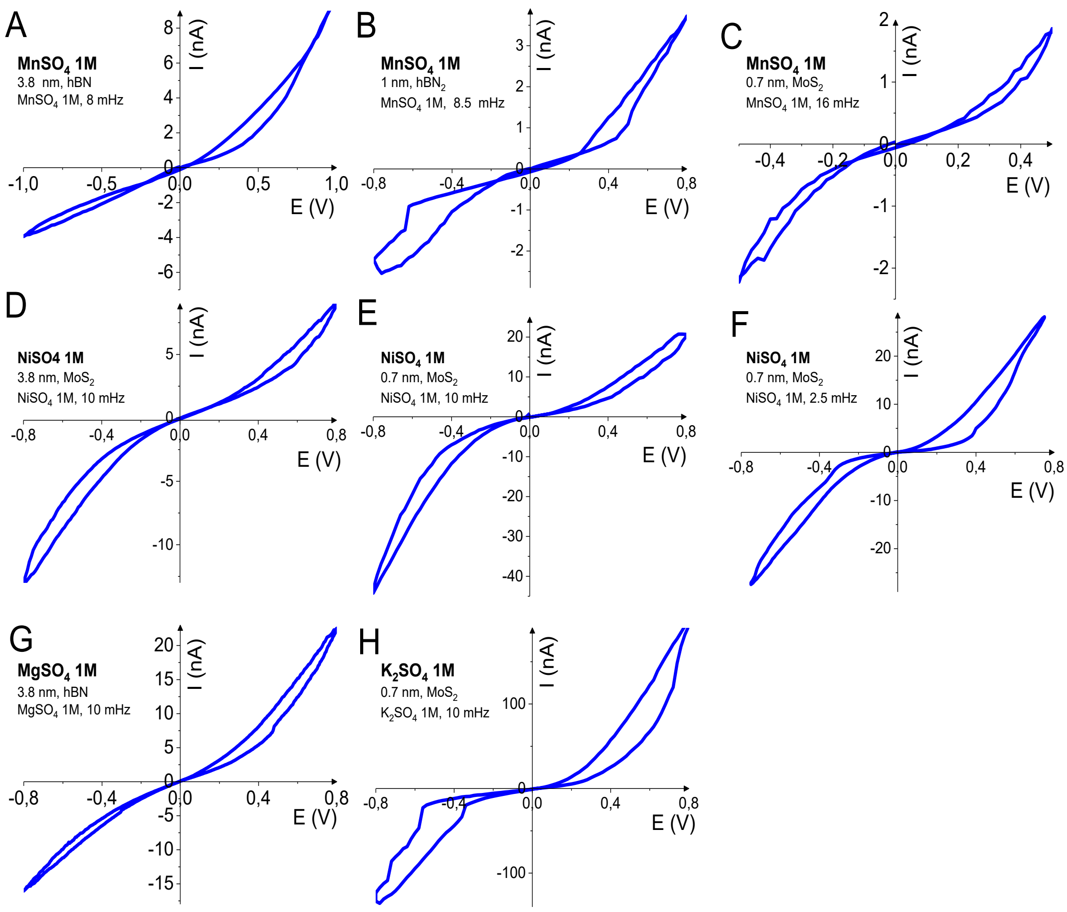

Supplementary Figure 24. Memristor effect using sulfate salts of different cations. Current-voltage characteristics of MoS_2 or hBN channels (see graph annotation) filled with different 1 M electrolytes composed of different cations (K^+, Mn^{2+}, Ni^{2+}, and Mg^{2+}). The heights of the devices, the salt and the frequency are written on each IV curve. Wien effect memristor is observed in all the above salts.

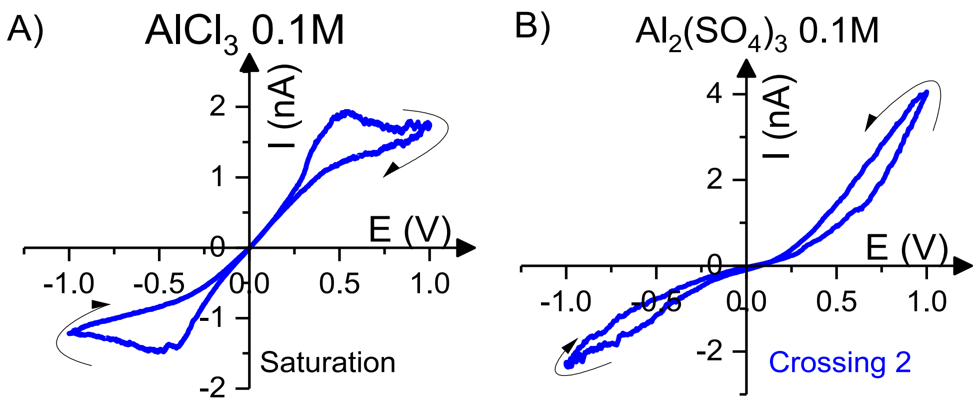

Supplementary Figure 25: Effect of aluminium salt anion on the memristor loop style. Current-voltage characteristics of hBN channels (h= 3.8 nm) filled with aluminium salt electrolytes having A) chloride or B) sulfate as counter ion under alternative triangular voltage of frequency ~2 mHz. The effect is shown at concentration of 0.1M of Al^{3+}.

10. Types of observed saturation memristors

Three distinct subtypes of external polarization— negative differential resistance (NDR), limiting current, and over limiting current (extended space charge)—were observed experimentally (Supplementary Figure 26). These behaviours provide insight into ion transport dynamics within nanoconfined systems and their response to varying voltages and ionic concentrations.

NDR is characterized by a decrease in conductance as voltage increases, leading to a reduction in current at higher voltages. This phenomenon can arise from multiple mechanisms. Siwy et al. [13] attributed NDR in asymmetric nanopores to interactions between ions and negatively charged pore walls, which reverse rectification direction. Bivalent ion binding and unbinding create fluctuations in the potential profile, enabling a flashing ratchet mechanism for ion transport. At specific ionic concentrations and voltages, these dynamics limit ion flow, resulting in the observed NDR effect. Lin et al. [14] proposed that NDR in nanopores could result from electro diffusio-osmosis, where electroosmotic and diffusio-osmotic flows compete near the pore opening. At high voltages, electroosmosis dominates, introducing low-conductivity solution into the pore and reducing ionic current. This mechanism is influenced by salinity gradients, pH-regulated surface charges, and pore geometry. Similarly, Ramirez et al. [15] linked NDR in conical nanopores immersed in KF solutions at low concentrations to fluoride ion accumulation and interaction with negatively charged pore walls, altering ionic conduction near the pore tip. In our experiments, NDR was predominantly observed at low frequencies and is likely due to ion-wall interactions, as electroosmotic effects are negligible in confined nanochannels [16, 17]. Yang et al. [18] expanded the understanding of NDR, demonstrating its emergence from ionic charge redistribution during hysteretic and rectified transport in conical nanopores. They identified dynamic enrichment and depletion of ions near the pore tip as key factors and showed that NDR can be tuned by manipulating ionic strength, nanopore geometry, and surface charge density.

In limiting current case, another subtype of external polarization, there are two regions: (I) an initial Ohmic region with a linear current-voltage relationship, and (II) a limiting current plateau where current saturates despite increasing voltage. This behaviour was experimentally observed at higher ionic concentrations and is explained by ion concentration polarization. Here, ion depletion at the nanochannel entrance forms a constant concentration gradient in the quasineutral electrolyte, limiting current flow to the Nernst diffusion value at high voltages [4, 19].

Overlimiting current, a third subtype, was observed at low ionic concentrations (e.g., 10 mM). It arises from extreme ion depletion at the channel entrance and the formation of extended polarized space-charge layers. These layers induce spontaneous convective mixing and vortex formation due to amplified local electric fields at high voltages, resulting in an increased slope in the current-voltage curve (region III) [20, 21]. These findings highlight the complexity of ion transport phenomena in nanoconfined systems and their dependence on ionic concentrations, applied voltages, and channel properties.

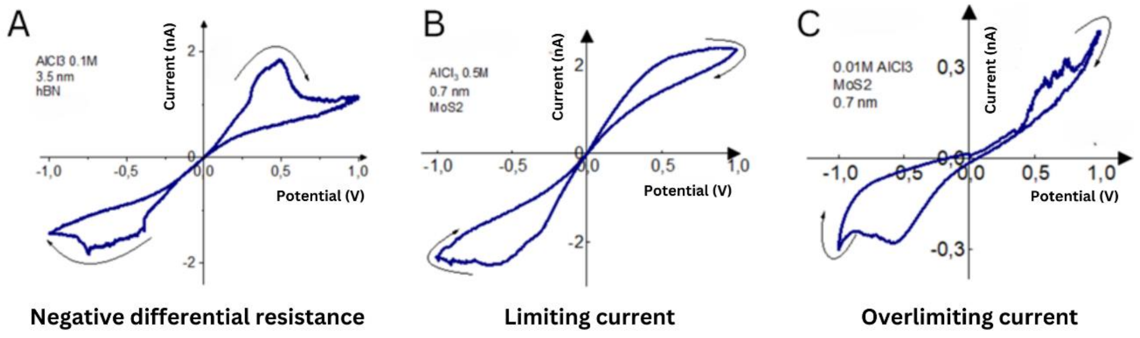

Supplementary Figure 26: Saturation memristor M2 observed in nanochannels. Three different types of saturation memristor A) negative differential resistance, B) limiting current, and C) Overlimiting current were observed, the experimental conditions are written on each curve.

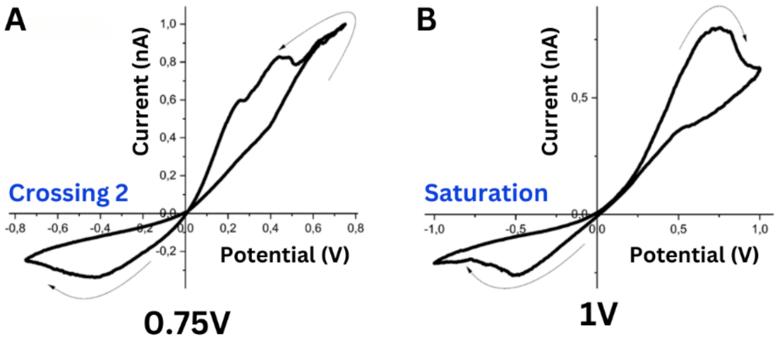

Supplementary Figure 27. Saturation memristor at high voltage. Alternating triangular voltage using different voltage ranges were applied on 0.7 nm hBN device using $AlCl_3$ 0.1M as electrolyte. The memristor effect Crossing 2 (M3) occurred at 0.75V, however, higher voltage application using same scan rate result in the appearance of saturation (M2) loop style. The M3 loopstyle indicate intrachannel conductance limitation while at higher voltage there is formation of depletion layer at the entrance of the nanochannel, which controls the further ion movement.

11. Endurance of memristors

One of the primary challenges with nanofluidic memristors is achieving a robust device capable of showing consistent memristive loop for numerous cycles. In this study, we evaluated the robustness of two memristive effects, saturation and Wien, up to hundreds of IV cycles. Both types of memristors demonstrated good reproducibility of the cycles, with a uniform range of conductances in both the on and off states, thereby yielding a consistent on-off ratio.

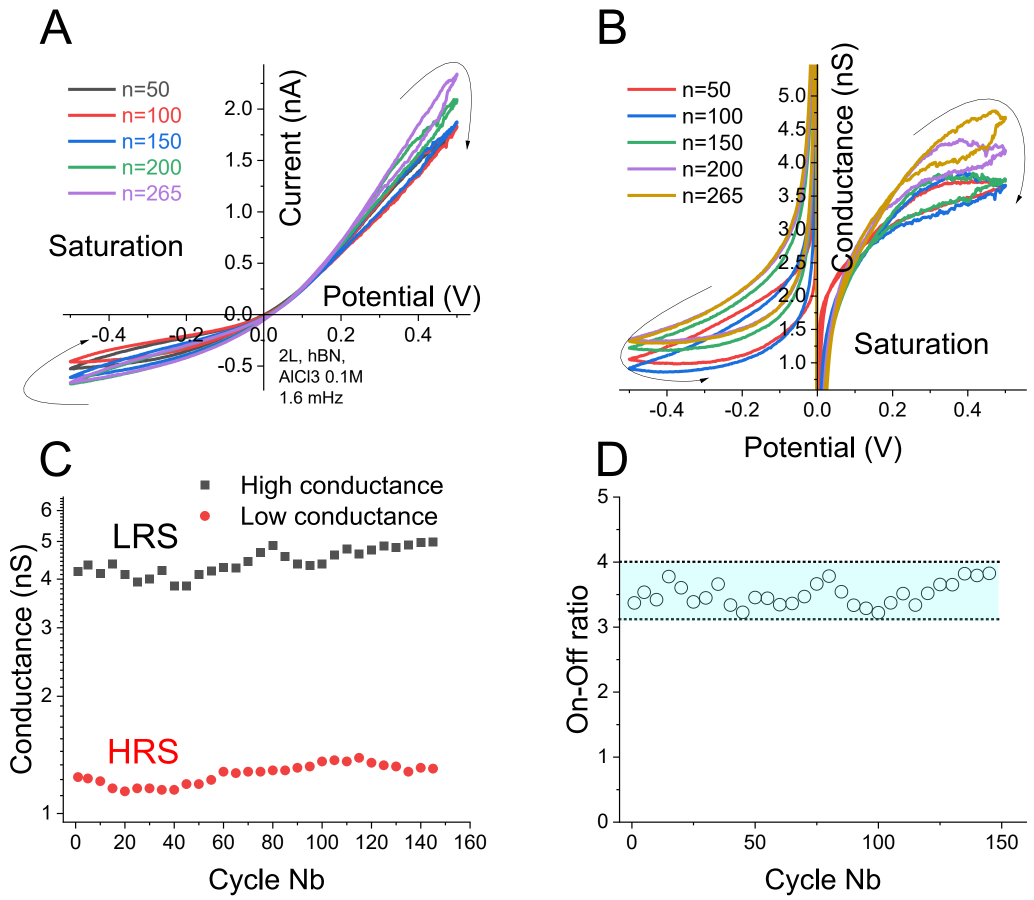

Supplementary Figure 28: Endurance of saturation memristor. A) Current-voltage characteristic and B) instantaneous conductance-voltage characteristic of 0.7 nm hBN device in $AlCl_3$ 0.1M electrolyte under 0.5 V and 2 mHz alternative triangular voltage. Saturation memristive effect (M1) is seen using these conditions. C) The low and high resistive states and D) the on-off ratio of conductance vs the number of alternative cycles.

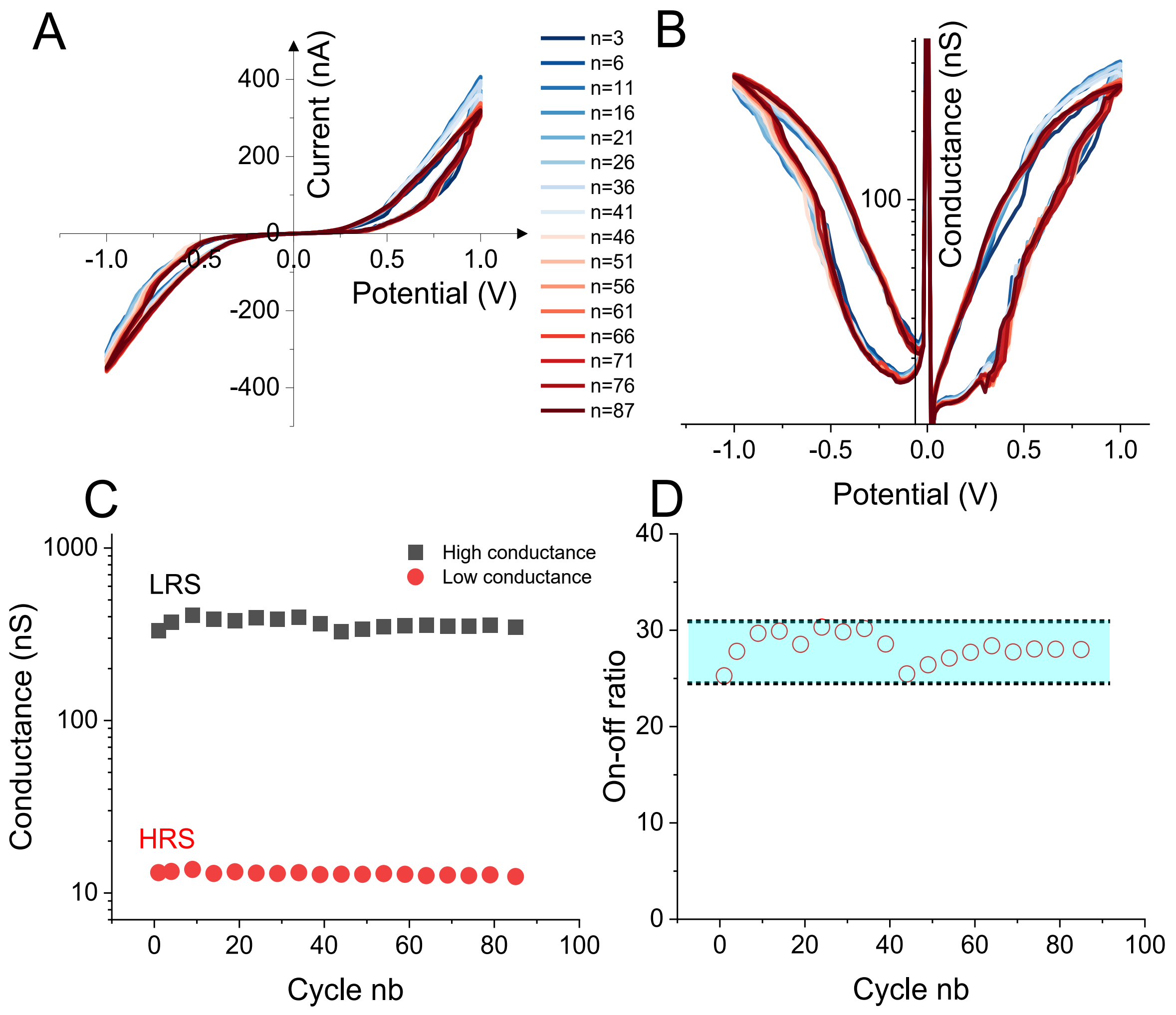

Supplementary Figure 29: Endurance of Wien memristor. A) current-voltage characteristic and B) instantaneous conductance-voltage characteristic of hBN device (h = 0.7 nm) in HCl 1M electrolyte under alternative triangular voltage. Memristive effect Wien is seen using these conditions. C) The low and high resistive states and D) the on-off ratio of conductance vs the number of alternative cycles.

12. Successive conductance strengthening and weakening

One potential future application for memristors is in neuromorphic computing, where they could serve as crucial components of circuits. The nanofluidic memristor could act similar to a synapse between two neurons, possessing a certain degree of plasticity. This means the conduction of the electrical signal could be strengthened (potentiation) or weakened (depression) through successive synaptic activations. Experimenting with successive positive or negative voltage cycles on the nanofluidic memristor could be intriguing in mirroring the plasticity of biological synapses, where the various conduction trends would be observed in different memristive styles. Previous demonstrations on activated carbon channels presenting crossing style memristor have shown that successive applications of positive (negative) voltage led to an increase (decrease) in the resulting current [3]. The results depicted in Supplementary figure 30 indicate that for Wien memristors, successive positive or negative potentials result in increased currents, thereby strengthening the synapse. Applying a high voltage pulse and reading at low voltage created a volatile memory, where the conductance returned to its initial value before the pulse application after a certain period (~5 minutes). This was verified through successive "writing" by applying short pulses of -400 mV and reading at -30 mV, while "erasing" occurred through relaxation of the device at zero volts. Erasing at zero volts, rather than

using pulses of opposite polarity as in the crossing memristor (Supplementary Figure 30C), was chosen because the memory here is volatile, and applying pulses of opposite polarity would result in further conductance increase due to more polyelectrolyte formation. The formation of polyelectrolyte depends on the magnitude of the applied potential rather than on its polarity.

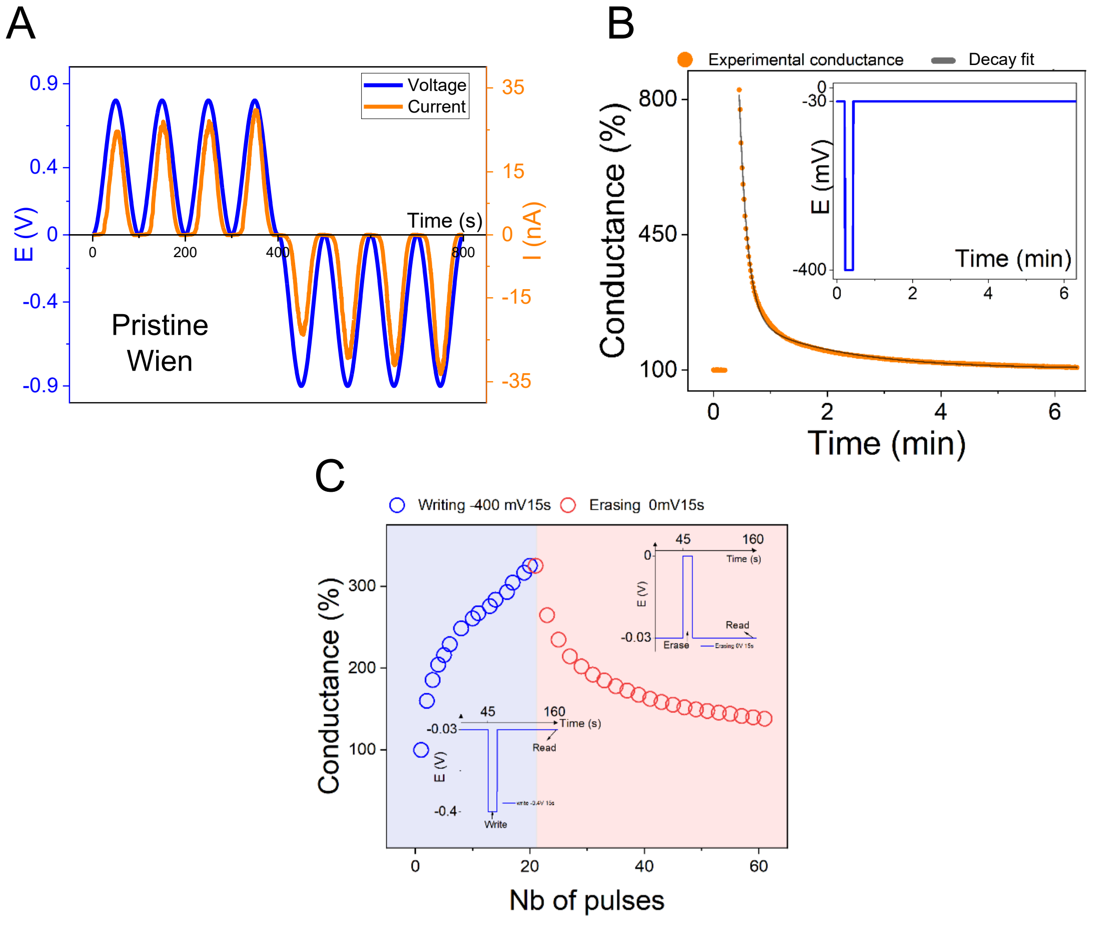

to c

Supplementary Figure 30: Programming a nanochannel through reversible conductance strengthening. A. Evolution of the ionic current (orange) under voltage pulses of constant sign (blue). Pulses result in an increase of conductance. B. Conductance change following a positive voltage pulse, exhibiting short (< 2 min) memory. The conductance is read by applying a weak square voltage wave that has no sensible impact on the state of the system, and modified through a strong voltage spike. Orange points are experimental data. The black solid line is a decay fit. Inset: applied voltage as function of time. The red arrow indicates the beginning of the voltage spike. C Successive pulses effect on the nanochannel's conductance. 20 write spikes (-0.4 V, 15 s) are applied, followed by 40 erase spikes (0 V, 10 s) which bring back the system to its initial state. Between each spike, the conductance is let to stabilize for two minutes and is then measured with a read pulse (-0.03 V). The hBN nanochannels A) h = 2 nm, B-C) 7 nm are measured with KCl 3 M (pH=2), which showed Wien effect memristor under these high salt and low pH conditions.

13. Relaxation at zero-volt in Wien effect

When a device operates continuously and is subjected to relatively high reading voltages, relaxing it to zero volts can enhance or suppress its conductance, depending on the memristive effect involved (refer to the main text for details). This paragraph focuses on the conductance-time characteristic

observed when relaxing a device that exhibits the Wien effect, across different relaxation times. It is noted that conductance suppression, both in MoS_2 and hBN devices, is less significant at short times and the suppression increases as the duration of relaxation at zero volts extends (Figure 6 and Supplementary Figure 31). The time required for the device to regain its conductance state before the relaxation also depends on the length of the relaxation period; shorter relaxation periods allow the device to return to its initial conductance state more quickly. These phenomena can be attributed to the time needed to dissociate the polyelectrolyte formed at high voltages during device relaxation and its subsequent reformation when high reading voltages are reapplied.

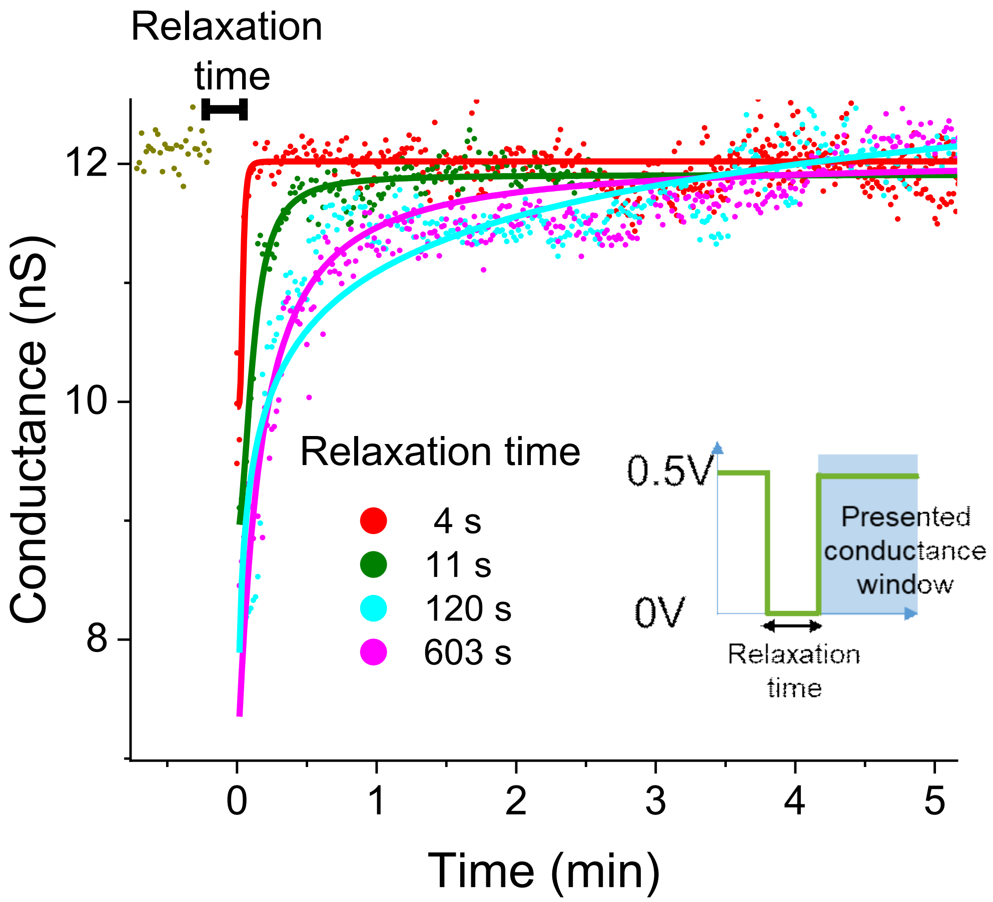

Supplementary Figure 31: Effect of Relaxation time on Wien memristor at high reading voltage. Graph showing the effect of the relaxation time (at 0V) on the conductance variation of hBN nanochannel (h = 2 nm) filled with KCl 1M electrolyte. Reading voltage 0.5 V, Erasing was done at 0V for different pulse durations. The return to the stable polyelectrolyte state is dependent on the relaxation time where shorter times resulted in faster recovery to the full polyelectrolyte state present at 0.5V.

14. Crossing 2 memristor

The writing, erasing, and relaxation processes of the crossing 2 memristor were explored, with variable parameters, including the amplitude of the writing and erasing potentials, and the duration of the writing pulse and relaxation at zero volts (Supplementary Figures 32-33).

An increase in the amplitude of the writing pulse resulted in higher conductance, whereas an increase in the erasing pulse magnitude led to more effective erasure. Thus, the conductance of the nanochannel can be precisely adjusted by the amplitude of the writing/erasing pulse, offering

potential benefits for analog computing and the emulation of synaptic plasticity. The application of positive polarity voltages leads to ion accumulation within the channels, thereby increasing conductance. In contrast, negative polarity voltages result in the depletion of counterions from the nanochannels.

Repeated application of pulses with the same magnitudes and durations led to a successive increase in the conductance of the nanochannels, eventually reaching a plateau. When erasing pulses were applied, the opposite effect was observed: the conductance returned to its initial value, and the synaptic weight (delta conductance) returned to zero (Supplementary Figure 32).

Furthermore, the conductance of the nanochannels can be controlled not only by the amplitude and number of pulses but also by the duration of pulsing. Conductance increases until reaching a maximum after pulses of 40 seconds in duration (Figure 6G). Notably, the duration of the pulse affects not only the increase in conductance but also its subsequent decay. Short pulses result in short-term memory, where the conductance decays to its original value after a few seconds. However, longer pulses lead to long-term memory, where the conductance remains stable. This is the first demonstration that short- or long-term memory can be achieved under the same experimental conditions by merely varying the duration of the programmed pulses. The long-term memory conductance value is also dependent on the pulse duration, with longer durations resulting in higher long-term conductance retention.

a) Voltage amplitude effect

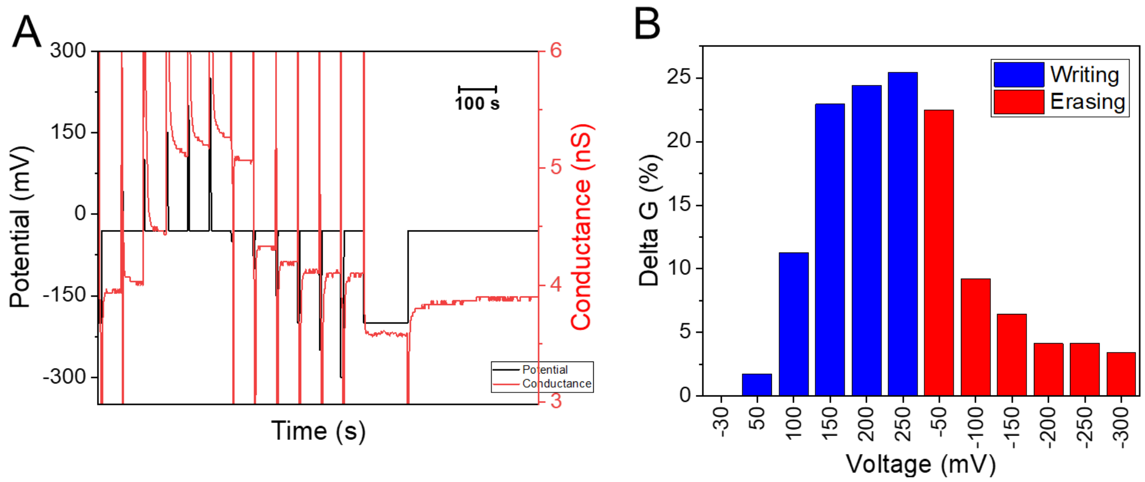

Supplementary Figure 32: Tuning the conductivity of crossing 2 memristor A) Current-time characteristic of 0.7 nm thick MoS_2 nanochannel filled with KCl 0.01M electrolyte. Reading voltage -0.03 V, writing and erasing pulses were applied for 5s with variable magnitudes as indicated in B.

b) Pulse duration effect

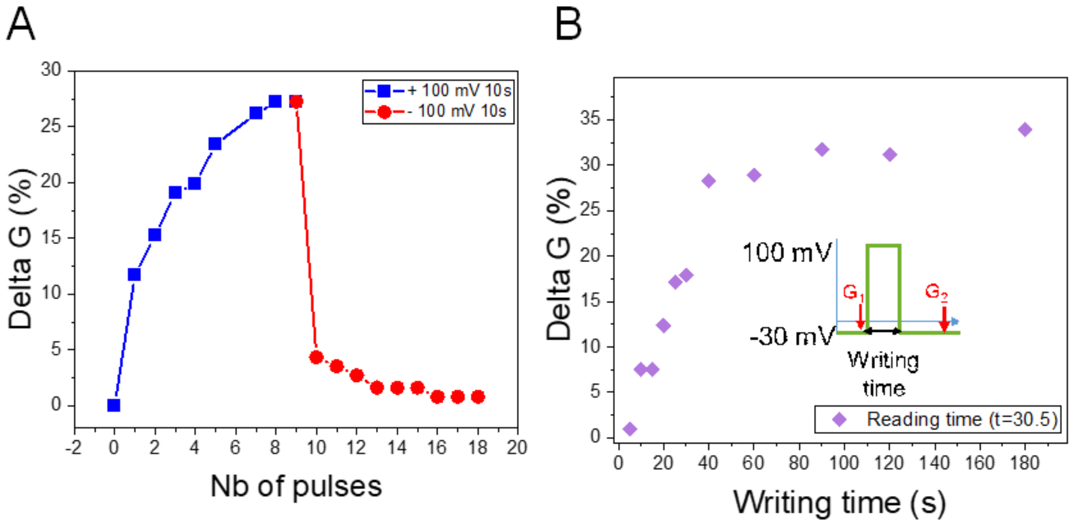

Supplementary Figure 33. Tuning the conductance of nanochannel of crossing 2 memristor. A) Strengthening and weakening of 0.7 nm channel filled with KCl 0.01M electrolyte by successive writing (and erasing respectively) pulses of +0.1 V (-0.1V respect.) for a duration of 10s. B) Increase of conductance depending on the duration of the writing where we can see saturation for durations higher than 60 s. The percentage change in conductance, ΔG (%), is calculated using the expression: $100 \times (G_2 - G_1) / G_1$, where G_1 and G_2 represents the conductance of the device before and after applying the write signal respectively.

15. Temperature effect

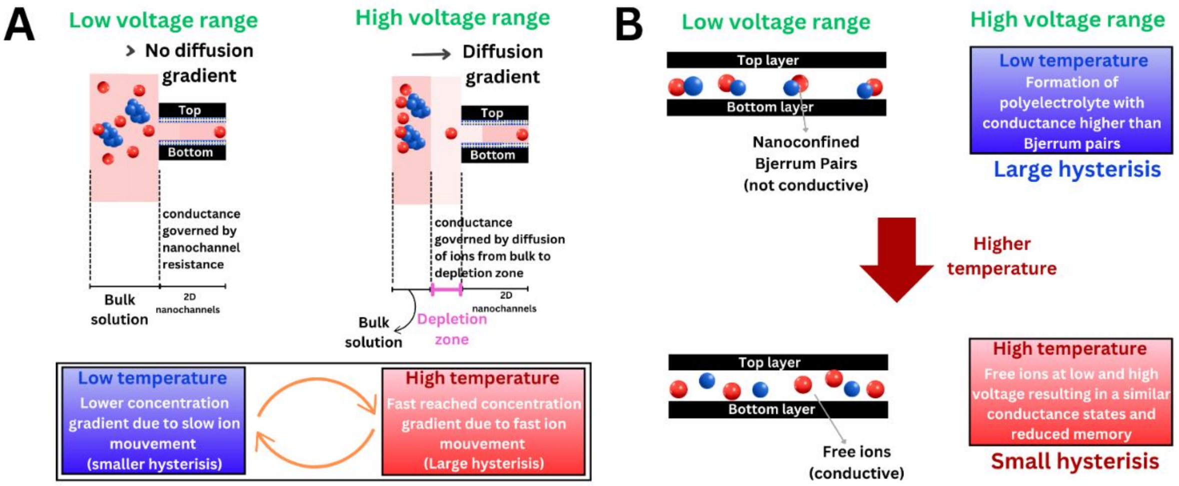

Supplementary Figure 34: Temperature effect on memristive behavior. A) Cartoon of the mechanism explaining the increase of hysteresis with temperature in saturation memristor (M2) where the depletion layer and concentration gradient is formed faster under higher temperature. B) The mechanism in Wien memristor (M4), where Bjerrum pairs transform to free ions under higher temperature and thus the existence of variable conductance states is decreased resulting in the decrease of hysteresis.

References

1. Bhardwaj, A., et al., *Fabrication of angstrom-scale two-dimensional channels for mass transport.* Nature protocols, 2024. **19**(1): p. 240-280.

2. Hu, S., et al., *Proton transport through one-atom-thick crystals.* Nature, 2014. **516**(7530): p. 227-230.

3. Robin, P., et al., *Long-term memory and synapse-like dynamics in two-dimensional nanofluidic channels.* Science, 2023. **379**(6628): p. 161-167.

4. Nam, S., et al., *Experimental verification of overlimiting current by surface conduction and electro-osmotic flow in microchannels.* Physical review letters, 2015. **114**(11): p. 114501.

5. Chua, L., *If it's pinched it'sa memristor.* Semiconductor Science and Technology, 2014. **29**(10): p. 104001.

6. Robin, P., N. Kavokine, and L. Bocquet, *Modeling of emergent memory and voltage spiking in ionic transport through angstrom-scale slits.* Science, 2021. **373**(6555): p. 687-691.

7. Radha, B., et al., *Molecular transport through capillaries made with atomic-scale precision.* Nature, 2016. **538**(7624): p. 222-225.

8. Goutham, S., et al., *Beyond steric selectivity of ions using ångström-scale capillaries.* Nature Nanotechnology, 2023.

9. He, Y., et al., *Tuning transport properties of nanofluidic devices with local charge inversion.* Journal of the American Chemical Society, 2009. **131**(14): p. 5194-5202.

10. Li, S.X., et al., *Direct observation of charge inversion in divalent nanofluidic devices.* Nano letters, 2015. **15**(8): p. 5046-5051.

11. Li, Y., et al., *Electrical Field Regulation of Ion Transport in Polyethylene Terephthalate Nanochannels.* ACS Applied Materials & Interfaces, 2019. **11**(41): p. 38055-38060.

12. Morikawa, K. and T. Tsukahara, *Shift of charge inversion point of a trivalent ion solution in a nanofluidic channel.* Colloid and Interface Science Communications, 2022. **50**: p. 100646.

13. Siwy, Z.S., et al., *Negative Incremental Resistance Induced by Calcium in Asymmetric Nanopores.* Nano Letters, 2006. **6**(3): p. 473-477.

14. Lin, C.-Y., et al., *Electrodiffusioosmosis-induced negative differential resistance in pH-regulated mesopores containing purely monovalent solutions.* ACS applied materials & interfaces, 2019. **12**(2): p. 3198-3204.

15. Ramirez, P., et al., *Negative differential resistance and threshold-switching in conical nanopores with KF solutions.* Applied Physics Letters, 2021. **118**(18).

16. Li, D., *Electroosmotic Flow and Electrophoresis in Nanochannels*, in *Electrokinetic Microfluidics and Nanofluidics*. 2022, Springer. p. 107-147.

17. Haywood, D.G., Z.D. Harms, and S.C. Jacobson, *Electroosmotic flow in nanofluidic channels.* Analytical chemistry, 2014. **86**(22): p. 11174-11180.

18. Yang, R., et al., *Negative differential resistance in conical nanopore iontronic memristors.* Journal of the American Chemical Society, 2024. **146**(19): p. 13183-13190.

19. Pu, Q., et al., *Ion-enrichment and ion-depletion effect of nanochannel structures.* Nano letters, 2004. **4**(6): p. 1099-1103.

20. Chang, H.-C. and G. Yossifon, *Understanding electrokinetics at the nanoscale: A perspective.* Biomicrofluidics, 2009. **3**(1).

21. Liel, U., et al., *Effect of field-focusing and ion selectivity on the extended space charge developed at the microchannel–nanochannel interface.* Journal of Physics: Condensed Matter, 2016. **28**(32): p. 324002.
